# Herb-target interaction network analysis helps to disclose molecular mechanism of traditional Chinese medicine

**DOI:** 10.1038/srep36767

**Published:** 2016-11-11

**Authors:** Hao Liang, Hao Ruan, Qi Ouyang, Luhua Lai

**Affiliations:** 1 Peking-Tsinghua Center for Life Sciences, Academy for Advanced Interdisciplinary Studies, Peking University, Beijing 100871, China; 2 Beijing National Laboratory for Molecular Sciences, State Key Laboratory for Structural Chemistry of Unstable and Stable Species, College of Chemistry and Molecular Engineering, Peking University, Beijing 100871, China; 3 State Key Laboratory for Artificial Microstructures and Mesoscopic Physics, School of Physics, Peking University, Beijing 100871, China; 4 Center for Quantitative Biology, Academy for Advanced Interdisciplinary Studies, Peking University, Beijing 100871, China

## Abstract

Though many studies have been performed to elucidate molecular mechanism of traditional Chinese medicines (TCMs) by identifying protein-compound interactions, no systematic analysis at herb level was reported. TCMs are prescribed by herbs and all compounds from a certain herb should be considered as a whole, thus studies at herb level may provide comprehensive understanding of TCMs. Here, we proposed a computational strategy to study molecular mechanism of TCM at herb level and used it to analyze a TCM anti-HIV formula. Herb-target network analysis was carried out between 17 HIV-related proteins and SH formula as well as three control groups based on systematic docking. Inhibitory herbs were identified and active compounds enrichment was found to contribute to the therapeutic effectiveness of herbs. Our study demonstrates that computational analysis of TCMs at herb level can catch the rationale of TCM formulation and serve as guidance for novel TCM formula design.

For thousands of years, traditional Chinese medicines (TCMs) are widely used in China for the prevention and treatment of diseases, especially the infectious and chronic ones. Different from western medicine, TCMs are usually prescribed in herbs, thus contain hundreds of compounds. Instead of binding to a single target protein with high affinity, TCMs may exert their effects on disease intervention through low affinity binding of multiple compounds to multiple different targets, leading to maximal therapeutic efficacy with minimal side effects. The holistic and synergetic nature of TCMs improves their performance on systems-level intervention of complex disease. However, it also brings a huge obstacle for the molecular understanding of TCMs therapeutic effectiveness by conventional pharmacology method. Merely elucidating individual protein-compound interaction pairs are insufficient to reveal the complex relationships between disease-related biological networks and TCM compound cocktails prescribed as herbs or their combinations.

With the development of systems biology and network pharmacology, many researchers attempted to study the molecular mechanisms of TCMs in systematic view^1^. Systematic docking and drug-target network analysis is widely applied in these studies^2^,^3^,^4^,^5^,^6^,^7^,^8^,^9^, however, the therapeutic effectiveness of TCM formulae is still ascribed to a few number of interactions between individual compounds and key target proteins. As TCMs are prescribed as herbs, compounds from one herb should be considered as a whole and mode of action at herb-level should be noticed. Apart from a few compounds with potent activity, other compounds may also contribute to the therapeutic effect by binding to the same or different disease related proteins. Thus, studies limited to compound level may only give limited understanding of molecular mechanisms of herbs and formulae, and the role an herb plays in disease intervention should be evaluated at herb level.

In addition, most TCMs formulae were designed by ancients based on experience and TCM principles, which may be limited when applied to newly emerged diseases, especially infectious diseases such as AIDS. Apart from a few examples, such as artemisinin^10^ and arsenic trioxide^11^, success in isolation of individual active agents is rare. Single-target drug discovery paradigm of TCMs is usually bottlenecked by weak binding affinity and bad specificity of TCM compounds. Therefore, methods for designing novel formulae or optimization of ancient formulae at herb level are in urgent need.

Human immunodeficiency virus (HIV) has become an unprecedented threat against global health. Among the two types of HIV identified, HIV-1 is much more virulent and widespread, and is considered as the main threat. The HIV-1 genome is composed of 9 genes and encodes 19 proteins, including enzymes (protease, reverse transcriptase and integrase), structural proteins (p7, p24 and p17, forming nucleocapsid, capsid and matrix, respectively), accessory proteins (Nef, Rev, Tat, Vif, Vpr and Vpu), and envelop protein, which is cleaved into two glycoproteins, gp120 and gp41. The entire replication cycle^12^ consists of thirteen steps, involving dozens of viral proteins and hundreds of host proteins^13^. The targets of available drugs, however, are limited, including only protease, transcriptase, integrase, gp41 and host protein CCR5^14^. Although they dramatically reduce the morbidity and mortality of AIDS, these drugs or their combinations^15^,^16^,^17^ (highly active antiretroviral therapy, HAART) suffer from rapidly emerged drug resistance mutations and side effects.

SH formula is a TCM anti-HIV-1 formula (also called “Si-Ai-Te-San” in Chinese) developed by combining TCM principles and experimental screening of more than 1000 TCM herbs^18^,^19^,^20^. It is composed of five herbs, including *Glycyrrhiza uralensis* (Gancao), *Carthamus tinctorius* (Honghua), *Astragalus membranaceus* (Huangqi), *Morus alba* (Sangbaipi) and *Artemisia capillaries* (Yinchenhao). *In vitro* experimental studies have validated its anti-HIV activity and identified *Morus alba* and *Glycyrrhiza uralensis* as the most potent anti-HIV-1 herbs with EC50 value as 14.3 μg/ml and 10.1 μg/ml, respectively^21^. SH formula was widely used for people living with HIV/AIDS in China and Southeast Asia countries, and clinical trials have been performed in Thailand since 1999. Clinical trials demonstrated that SH formula is non-toxic and able to decreased HIV viral load in 14–35% of HIV-positive patients when used alone^22^. In addition, combination treatment of SH formula and nucleoside analog reverse transcriptase inhibitors (NRTIs) exhibited greater antiviral activity than NRTIs alone^23^. It was also reported that the combination of SH formula and protease inhibitor atazanavir enhances their inhibition potentials^24^. SH formula has been approved by the Ministry of Public Health of Thailand for clinical use. However, the inhibition mechanism remains elusive, which severely impedes in-depth understanding and further optimization of SH formula.

In the present study, we proposed an herb based strategy combining systematic docking and herb-target network analysis to study TCM formulae at herb-level. As a concrete example, we have applied this strategy to understand why SH formula is effective against HIV-1 and to reveal its design principles. Active herbs were identified and target-herb interaction maps were analyzed. Inhibitory herbs were characterized as containing high enrichment of active compounds rather than single potent inhibitor. Our study provides a novel way to study molecular mechanisms of TCMs and may facilitate rational design or optimization of TCM formulae against complex diseases.

## Results

### Herb based strategy to study TCM formulae

**Figure 1:**
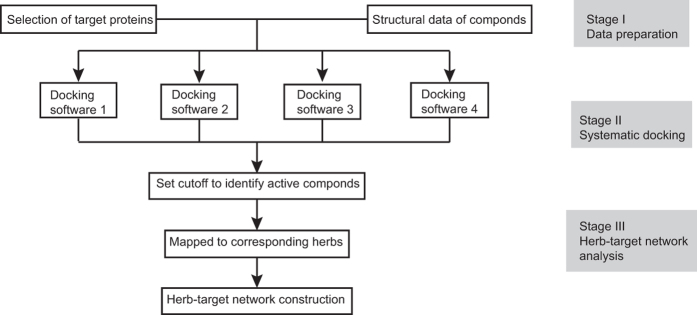
The flow chart of herb based strategy.

The herb based strategy to understand the molecular mechanism of TCM formulae at herb level includes three stages (as shown in Fig. 1). The first stage deals with data preparation. For the TCM formula of interest, compounds in all the component herbs should be listed first by searching existing TCM databases or experimental studies and be modeled for their three-dimensional (3D) structures. For the corresponding disease that the TCM formula treats, all targets with 3D structures experimentally determined or modeled are collected. In the second stage, connections between the compounds and the targets are built based on systematic docking. A certain scoring cutoff and RMSD restriction are set to distinguish active and inactive compounds. In the third stage, the herb-target network is constructed by mapping active compounds to their corresponding herbs and analyzed. To quantitatively evaluate the effectiveness of herb target interaction, we define the herb-target factor (HTF) as follows:


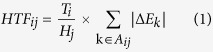


where *HTF*_*ij*_ is the HTF of herb *i* against target protein *j*, *A*_*ij*_ is the active compound set of herb *i* against target protein *j*, Δ*E*_*k*_ is the docking score of active compound *k*, *T*_*i*_ is the total target number of herb *i*, *H*_*j*_ is the number of herbs that target protein *j*. For a given herb-target interaction, the effectiveness is not only related to the sum of binding affinity of active compounds, but also affected by the ability of herbs to inhibit multi-targets. Besides, if a protein has already been targeted by several herbs, this herb-target interaction is less important and can be compensated by the inhibition of other herbs. The greater value of HTF indicates stronger and more critical herb-target interaction. Finally, based on the herb-target network and HTF analysis, we can identify active herbs and their potential targets and understand the molecular mechanism of TCM formulae at herb level.

### Application of the herb based strategy to reveal molecular mechanism of the SH formula

#### Data preparation

The replication cycle of HIV-1 is complicated, involving 15 viral proteins as well as hundreds of host proteins^13^. With plenty of available structures for most HIV-1 related proteins, HIV-1 is a suitable system for herb based study. For simplicity, we only focus on viral proteins and the most well studied host proteins, including ten viral proteins protease (PR), reverse transcriptase (RT), integrase (IN), p7 (NC), p24 (CA), p17 (MA), Nef, Vpr, gp120, gp41 and four host proteins including CCR5, CXCR4, Cyclin T1 (CycT1) and elongin C (ELOC). For RT, CA and Nef, multiple structures with different binding ligands accommodated in different binding pockets have been solved. To thoroughly investigate the possible molecular mechanism of SH formula, all structures of these proteins were used for molecular docking. Thus, a total of 17 target protein structures are used for molecular docking.

For efficient compound extraction from TCM herbs, we first constructed a novel subset of TCM database, named TCM herb database (TCMHD) according to herb information from Chinese Pharmacopoeia (2010 Edition) and 3D structures from Traditional Chinese Medicine Database (TCMD, NeoTrident Co., Ltd.)^25^,^26^. The herb information from TCMD was carefully checked to make sure they are exactly identical with those from Chinese Pharmacopoeia. Some herbs have not been exhaustively studied by photochemistry method, thus only a few compounds are listed in TCMD. For simplicity, we only focused on the fully studied herbs with at least ten compounds identified. In addition, the following criteria were applied to filter and modify all TCMHD compounds: (1) compounds composed of only carbon and hydrogen atoms were removed due to their poor water solubility and (2) considering the fact that glycosides are prone to hydrolysis *in vivo*, glycosyl moieties were removed and replaced with hydrogen atoms. Finally, a total of 272 non-redundant herbs and 4851compounds were used to construct TCMHD.

Based on TCMHD, we were able to get compounds for all the herbs in SH formula. We obtained 84, 32, 16, 47 and 54 compounds from *Glycyrrhiza uralensis*, *Carthamus tinctorius*, *Astragalus membranaceus*, *Morus alba* and *Artemisia capillaries*, respectively. After removing the repeated compounds from different herbs, the total compound number of SH formula is 226.

In order to compare SH formula with other non HIV treating formulae or random selection of TCM compounds, we designed three control groups. The first control group consists of 226 compounds randomly selected from TCMHD, irrespective of the herbs they belong to, which was named as all random (AR) group. The second control group contains 226 compounds randomly selected from TCMHD but sharing similar chemical properties distributions with SH formula (SP group), as shown in Supplementary Fig. S1. The third control group (XFZY group) contains compounds from a non-HIV related prescription: the Xuefuzhuyu formula, an ancient prescription for treating cardiovascular disease for over 200 years. 229 compounds were collected from seven Xuefuzhuyu formula herbs to constitute XFZY group, including, *Bupleurum chinense* (Chaihu), *Ligusticum wallichii* (Chuanxiong), *Paeonia rubra* (Chishao), *Angelica sinensis* (Danggui), *Rehmannia glutinosa* (Dihuang), *Platycodon grandiflorum* (Jiegeng) and *Achyranthes bidentate* (Niuxi).

#### Systematic docking

We used three widely accepted docking software to depict the relationship between HIV-1 related proteins and TCMHD compounds, including AutoDock 4.2^27^, AutoDock Vina^28^ and Glide 6.9. For Glide 6.9, both standard precision (SP)^29^ and extra precision (XP)^30^ were employed. Previous studies have validated the reliability of all these docking tools to select active compounds against HIV-related proteins^31^,^32^. Moreover, it was reported that the combination of different docking software and scoring functions may improve the accuracy of molecular docking and hit rate of virtual screen^33^,^34^. Thus, we performed systematic docking between 4851 TCM compounds and HIV-1 related proteins, including 13 viral proteins and 4 host proteins by all four docking tools and integrated the docking results to distinguish potential active compounds. For each docking results, only the poses with the best score are subjected to further analysis. Compounds ranked as top 40% by all four docking simulations were extracted, and their docking poses against certain protein pocket generated by different docking software are compared. If one compound is predicted to bind with one protein pocket in similar mode (RMSD < 2 Å) by at least three docking software, it is considered as active compounds. Although the concentrations of different compounds in herbs vary, all compounds were treated as the same concentration for simplicity due to the lack of reliable concentration data. The ability to intervene HIV-1 replication of SH formula, AR group, SP group and XFZY group is firstly evaluated by the active compound numbers in each group, as listed in Table 1.

SH formula was predicted to interfere HIV-1 replication cycle by inhibiting 13 of all 17 HIV-1 related proteins. The great number of active compounds and ability to bind multi-targets validate the role that SH formula plays as potent anti-HIV-1 remedy. Similar with western medicine, viral enzymes and co-receptors are important and function as the targets for nearly 1/3 of all active compounds. In addition, there are also five, three and six compounds interacting with structural proteins NC, CA1 and CA2, breaking the assembly of viral nucleocapsid and capsid, respectively. Another process that SH formula may be involved is the initial contact between gp120 and CD4. Together with CCR5 and CXCR4 inhibitors, SH formula may block the viral replication cycle from the entry step. Five active compounds are found to possibly interfere with the interaction between ELOC and Vif, which impede the Vif-induced APOBEG3G degradation and liberate APOBEG3G for the cell protection. Finally, as the one of the most attractive target, CycT1 is inhibited by eight compounds, breaking interaction between Tat and cyclin T1 and deactivating transcription process of HIV.

As control groups, AR group and SP group are composed of compounds randomly selected from TCM database but sharing different chemical properties distribution (Supplementary Fig. S1). The number of active compounds in AR group and SP group are similar. Given the randomness of AR group, we expected that they should have little anti-HIV-1 activity. Indeed, SH formula outperform AR group for most target proteins. However, both AR and SP groups also contain a number of potentially active compounds. This implies that binding affinities at compound level are not sufficient to understand TCM activity. Considering that TCMs are prescribed as various herbs rather than individual compounds, we should re-think about the docking results at herb level. As a randomly selected herbs combination not targeting HIV, the XFZY group contains more herbs and more ingredients, however, both the target proteins number and active compounds number are much smaller than those of SH formula, as shown in Table 1. Thus, herb level analysis of individual herbs in SH formula and control groups are necessary and may provide a more comprehensive understanding of TCMs study.

#### Herb-target network analysis

**Figure 2:**
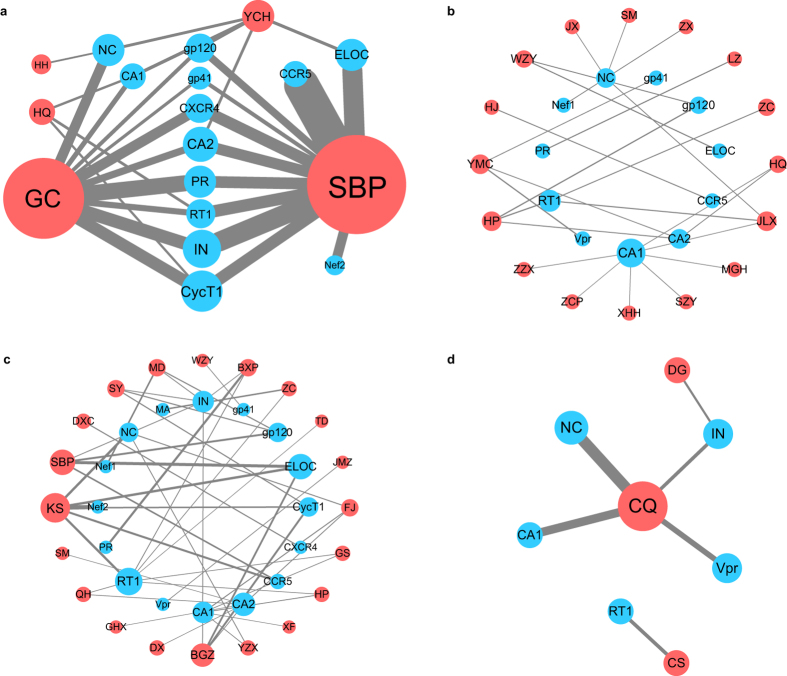
Herb-target network of HIV-1 related proteins and SH formula (**a**), AR group (**b**), SP group (**c**) and XFZY group (**d**). GC, HH, HQ, SBP, YCH, CQ, CS, DG represents *Glycyrrhiza uralensis*, *Carthamus tinctorius*, *Astragalus membranaceus*, *Morus alba*, *Artemisia capillaries*, *Ligusticum wallichii*, *Paeonia rubra*, and *Angelica sinensis* respectively. The Latin names for corresponding herbs in AR group (**b**) and SP group (**c**) are listed in Supplementary Table S2 and S3, respectively. Node sizes of herbs and targets are weighted by active compound numbers, edge sizes are weighted by HTFs.

To understand mode of action of SH formula at herb level, we mapped all the active compounds predicted back to their corresponding herbs and constructed the herb target network for the SH-HIV system, as well as for the three control groups. HTFs were calculated for each individual herb-target interaction, and the results were shown in Supplementary Table S1–4. Previous study suggested Glide XP has advantages in generating the greatest number of ligands with a reliable docking pose and lowest average RMSD when compared with crystal structure^34^. Thus, Glide XP score were used for the calculation of HTFs. The herb-target networks were plotted by using the number of active compounds corresponding to each herb or target as node sizes and HTF values as edge widths (Fig. 2).

As shown in Fig 2a, all SH formula herbs are predicted to be involved in HIV intervention by interacting with single or multiple target proteins. Both *Glycyrrhiza uralensis* and *Morus alba* are able to inhibit viral enzymes (PR, RT1 and IN), glycoproteins (gp120 and gp41) and two host proteins (CycT1 and CXCR4). *Glycyrrhiza uralensis* exhibits inhibition effect against viral structural proteins, like CA1 and NC, while *Morus alba* is inclined to deal with virus entry process (CCR5) and regulatory proteins (ELOC and Nef2). On the contrary, *Carthamus tinctorius*, *Astragalus membranaceus*, *Artemisia capillaries* of SH formula and *Ligusticum wallichii*, *Paeonia rubra*, *Angelica sinensis* of XFZY group (Fig. 2d) only show minor effect in HIV intervention as they interact weakly with few target proteins.

Although both the number of target proteins and active compounds of AR group and SP group are similar with those of SH formula, the interaction of each herb-target pair within AR-HIV (Fig. 2b) and SP-HIV (Fig. 2c) network is much weaker. Besides, none of AR group and SP group herbs is capable to play critical role in intervention of HIV-1 replication network as *Glycyrrhiza uralensis* and *Morus alba* do. The active compounds distribution pattern seems to be distinct between AR/SP group and SH formula. To quantitatively analyze the role each herb played in HIV-1 intervention, we calculated the weighted degree centrality (*wC*_*D*_) of each herb-target network as follows:


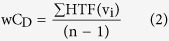


in which n is the node number of network, ∑HTF(v_i_) is the sum of HTFs for all edges connect to the node i. Unlike conventionally used degree centrality (C_D_), the node is re-weighted by the interaction between herb and target protein. Thus, the role of each herb is evaluated by both the ability to inhibit multiple targets but also the degree of inhibition. The calculated *wC*_*D*_ for SH formula herbs are 30.98, 0.09, 1.16, 59.92 and 3.11 for *Glycyrrhiza uralensis*, *Carthamus tinctorius*, *Astragalus membranaceus, Morus alba* and *Artemisia capillaries*, respectively. Our docking results and herb-target network analysis suggest that *Glycyrrhiza uralensis* and *Morus alba* are the most potent herb and contribute most of efficacy in SH formula. Our finding is consistent with *in vitro* experiment, which shows that *Morus alba* and *Glycyrrhiza uralensis* are the most potent anti-HIV-1 herbs with EC50 values of 14.3 μg/ml and 10.1 μg/ml, respectively^21^.

We then calculated *wC*_*D*_ for individual herb from AR group network and SP group network. However, the highest *wC*_*D*_ is only 1.13 (*Magnolia officinalis*, HP) and 3.72 (*Sophora flavescens*, KS) for AR group and SP group, respectively, which is far less than that for SH formula (59.92), indicating the inhibition is centralized on a few pivotal nodes in SH formula and scattered on hundreds of nodes in AR group and SP group. Obviously, to achieve similar inhibitory effect, the required herb numbers of AR group and SP group are much larger, and the inhibitory effect of each individual herb is far less than that of *Morus alba* and *Glycyrrhiza uralensis*, leading to the situation that patients need to take dozens or even hundreds of different herbs, while a remedy composed of so many herbs may bring tremendous inconvenience and potential toxicity.

#### Active compounds are enriched in SH formula

**Figure 3:**
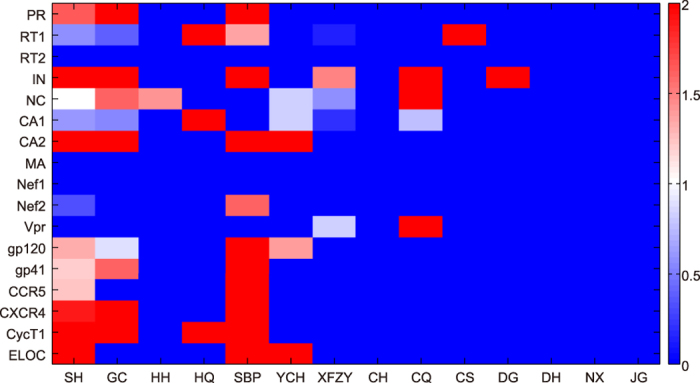
Heat map of EFs of SH formula (SH), *Glycyrrhiza uralensis* (GC), *Carthamus tinctorius* (HH), *Astragalus membranaceus* (HQ), *Morus alba* (SBP), *Artemisia capillaries* (YCH), XFZY group (XFZY), *Bupleurum chinense* (CH), *Ligusticum wallichii* (CQ), *Paeonia rubra* (CS), *Angelica sinensis* (DG), *Rehmannia glutinosa* (DH), *Platycodon grandiflorum* (JG), and *Achyranthes bidentata* (NX) against HIV-1 related proteins.

To further investigate the active compounds distribution at herb level, we used enrichment factor (EF) to evaluate the enrichment degree of active compounds. This enrichment factor is calculated as following:


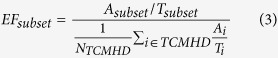


where *A*_*subset*_ and *T*_*subset*_ is the active compound number and total compound number of each subset, respectively, *N*_*TCMHD*_ is the herb number of TCMHD, *i* is the herbs of TCMHD, *A*_*i*_ and *T*_*i*_ is the active compound number and total compound number of each herb in TCMHD, respectively. If a subset has EF greater than 1 for a given protein, this subset is regard to be rich in active compounds and exhibit potential inhibition effect against this protein. To understand the anti-HIV-1 performance of randomly selected herbs, we calculated the EFs for SH formula, XFZY formula and individual herbs within them, as displayed in Fig. 3 and Supplementary Table S5.

Enrichment analysis suggests that active compounds are heavily distributed in SH formula, especially in *Morus alba* and *Glycyrrhiza uralensis*. On the contrary, EFs of XFZY group against almost all proteins are less than 1. Combining the docking results of SH formula and control groups, we conclude that the efficacy of SH formula comes from both the abundance and enrichment of active compound in certain inhibitory herbs, such as *Morus alba* and *Glycyrrhiza uralensis*.

Our study demonstrated that compound level analysis of TCMs may limit our understanding on the mode of action of TCM formulae. Multi-targets molecular docking and herb-target network analysis may be useful in TCMs formulae mechanism studies and in identifying novel inhibitory herbs. Except for binding affinities of individual compounds, the abundance and enriched distribution of active compounds in specific herbs are also need to be taken into consideration in TCMs studies.

## Discussion

Many studies have been reported to elucidate the molecular mechanism of TCM formulae in systematic view^2^,^3^,^4^,^5^,^6^,^7^,^8^,^9^. Compound-target networks were constructed and analyzed in these studies, and the effectiveness of TCM formulae was evaluated at compound level. However, our study suggested that compounds randomly selected from TCM database exhibit similar binding affinities but distinct degree centrality and distributions when compared with compounds from anti-HIV formula. Thus, studies limited to compound-target network may only give limited understanding and insufficient to elucidate complicate mode of action of TCM herbs and formulae. Analysis at herb level is worthwhile and may explain the mechanism of SH formula from a novel and comprehensive perspective.

Though widely accepted in drug design studies, each docking tools we used has its own set of biases. To avoid the potential biases of single docking tool may introduce, researchers tried to combine the docking results of several docking tools. For example, Tuccinardi *et al*. evaluated the reliability of a consensus docking protocol for three different targets of the Directory of Useful Decoys (DUD) using the combination of ten different docking tools, including AutoDock, DOCK, FRED, Glide SP, Glide XP, Gold with GoldScore, ChemScore, Astex Statistical Potential, ChemPLP, and AutoDock VINA^34^. The docking results were clustered using RMSD threshold as 2 Å. Their studies indicated that with regard to the AUC, in all three cases the consensus docking showed good results with values ranging from 0.67 to 0.90 and this approach performed as one of the best available methods found in the literature. Therefore, though the biases of docking software do exist, the consensus docking strategy provides more reliable results for the understanding of compound-target relationship and construction of herb-target network.

Compared to only using one docking program, requiring the selected compounds in a certain top ranking (e.g. 40%) of all the four docking tools is more demanding. We have tested the influence of different cutoff settings. When the cutoff was set to top 10% and top 20%, only very few active compounds were identified (Supplementary Table S6 and S7), from which no useful information can be extracted for further analysis. With higher (top 30%) or lower (top 50%) cutoff, the number of compounds identified as active compounds varies (Supplementary Table S8 and S13). However, no matter what cutoff we use, the active compounds identified within SH formula, AR group and SP group are similar against both individual target proteins or the whole HIV network, indicating the disadvantage of compound level analysis and the necessity to apply herb-target network analysis in TCM studies. The herb-target network analysis of SH formula at top 30% (Supplementary Table S9, Supplementary Fig. S2) or top 50% (Supplementary Table S14, Supplementary Fig. S3) cutoff shows that *Glycyrrhiza uralensis* and *Morus alba* are still the most potent inhibitory herb and able to interact with most of target proteins. The inhibition against HIV network of AR group (Supplementary Table S10, S15, Supplementary Fig. S2, S3) and SP group (Supplementary Table S11, S16, Supplementary Fig. S2, S3) is scattered by weak interaction of dozens of herbs. Some herbs in control groups are able to interact with multiple target proteins. However, the HTFs are much smaller when compared with those of SH formula herb. Thus, the herb-target network analysis is robust and independent of cutoff setting.

In the previous reported drug discovery studies of TCMs, the screen of TCM agents is still focused on the identification of potent compounds. Most previous experimental or computational studies tried to isolate single or multiple active compounds from herbs and ascribe the efficacy to these identified compounds. This kind of method is suitable for conventional western drug development and have contributed much for drug discovery^35^. However, the difficulties in experimental isolation of active compound as well as the weak binding affinity have become huge obstacles for the development of TCMs. On the other hand, disease intervention can be effectively reached by simultaneously perturbing multiple targets^36^,^37^, for which TCMs have advantages as mixtures of herbs containing many compounds. As TCMs are usually prescribed and taken in the form of herbs, the identification of active herbs rather than active compounds is more reasonable and feasible. In addition, though most of the currently used TCM formulae have proved their efficacy for long existing diseases, formulae development for newly emerged diseases remains challenging. To design novel formula against a newly emerged disease, a number of effective herbs and their combination need to be found. Our study suggests that the efficacy of TCM activity comes from both abundance and enrichment of active compounds. Thus, with the help of systematic docking and subsequent herb-target network analysis, we may be able to identify herbs rich in active compounds. By combination of several active herbs against different disease related proteins, novel formulae can be designed rationally with clear molecular mechanism.

## Methods

### Target protein selection

For viral proteins and the most well studied host proteins, we choose proper structures, as shown in Table 2. Ten viral proteins are structurally available, including protease (PR), reverse transcriptase (RT), integrase (IN), p7 (NC), p24 (CA), p17 (MA), Nef, Vpr, gp120 and gp41. All these protein structures were solved by X-ray crystallography with high resolution except for NC and Vpr, which were solved by NMR due to their high flexibility. For RT, CA and Nef, multiple structures with different binding ligands accommodated in different binding pockets have been solved. To thoroughly investigate the possible molecular mechanism of SH formula, all structures of these proteins were used for molecular docking. Four host proteins that play critical role in virus replication cycle were regarded as target protein, including CCR5, CXCR4, Cyclin T1 (CycT1) and elongin C (ELOC), whose binding partner is gp120, Tat and Vif, respectively. The crystal structure of CCR5 with maraviroc, CCR4 with antagonist IT1t and ELOC with Vif have been solved. However, the structure of CycT1 in complex with Tat and transactivation-responsive (TAR) RNA is unknown. Using complex structure of *Equus caballus* CycT1 (eCycT1) structure in complex with Equine infectious anemia virus (EIAV) Tat/TAR RNA (PDB ID 2W2H)^38^,^39^ as template, we constructed *Homo sapiens* CycT1 (hCycT1) and HIV-1 Tat-TAR RNA complex structure using Modeler software^40^. Considering the high sequence identity (98%) between eCycT1 and hCycT1, the constructed structure is reliable for docking.

### Construction of TCM herb database

TCM database is a comprehensive database containing ~23000 compounds from ~7000 TCMs. Some TCMs are parts of animals, minerals or poorly known herbs. Our aim is to construct a novel subset of TCM database consisting of compounds from only widely accepted herbs. According to the Chinese Pharmacopoeia (2010 Edition), there are 500~600 herbs that are widely acknowledged and suggested for clinical use. Thus, we extracted the information of these herbs from Chinese Pharmacopoeia, including Latin names, Chinese name and edible parts. Then, herbs from TCM database with identical information were selected together with their corresponding compounds. The compounds number criterion was set to ten to remove the poorly studied herbs. Duplicate compounds, hydrocarbon compounds were removed and glycosyl moieties were replaced with hydrogen atoms. Finally, a total of 272 non-redundant herbs and 4851 compounds were used to construct TCM herb database. The Latin name, Chinese name and the compounds number of TCM herb database are listed in Supplementary Table S18.

### Construction of control groups

The AR group are composed of 226 compounds randomly selected from TCMHD without any restriction in chemical properties and irrespective of the herbs they belong to. There is no overlapping compound between AR group and SH formula (Supplementary Fig. S4).

The SP group was set to explore the relationship between chemical property distributions and anti-HIV-1 activities, so we need to make sure the chemical property distributions of compounds in this control group is similar with that in SH formula. Based on this principle, for each SH formula compound (seed compound), the following steps were performed: (1) calculate six chemical properties including number of hydrogen acceptors (HAs), number of hydrogen donors (HDs), molecular weight (MW), number of rings (RGs), number of rotatable bonds (RBs) and logP (LP) by XlogP3 software^41^; (2) chemical property ranges were set by defining MW range as ±50 and HAs/HDs/RGs/RBs/LP range as ±1; (3) TCMHD compounds satisfying all chemical property ranges were considered as similar compounds and a compound pool consisting of these similar compounds were constructed; (4) random selection was performed against similar compound pool. Finally, similar compounds for each SH formula compounds were collected to form the SP group. The chemical properties of some SH formula compounds are unique, thus there may be only one compound in the corresponding pool. For those compounds, SH formula compounds themselves are selected for the construction of SP group. There are totally 18 compounds overlapped between SH formula and SP group.

The third control group compounds come from Xuefuzhuyu formula, an ancient prescription which has been used to treat cardiovascular disease for over 200 years. The formula consists of eleven herbs, including *Glycyrrhiza uralensis* (Gancao), *Carthamus tinctorius* (Honghua), *Bupleurum chinense* (Chaihu), *Ligusticum wallichii* (Chuanxiong), *Paeonia rubra* (Chishao), *Angelica sinensis* (Danggui), *Rehmannia glutinosa* (Dihuang), *Platycodon grandiflorum* (Jiegeng), *Achyranthes bidentata* (Niuxi), *Prunus persica* (Taoren) and *Citrus aurantium* (Zhike). The molecular mechanism of Xuefuzhuyu formula has been investigated, and the target protein was reported to be cardiovascular related proteins^4^. Except for *Glycyrrhiza uralensis* and *Carthamus tinctorius*, compounds from the rest of Xuefuzhuyu formula should have no or little relation with HIV-1 related proteins. In addition, *Prunus persica* and *Citrus aurantium* contain only three and nine compounds and are not included in TCMHD. Therefore, we set the third control group, XFZY group, by collecting 49, 58, 9, 51, 63, 12 and 12 compounds from *Bupleurum chinense*, *Ligusticum wallichii*, *Paeonia rubra*, *Angelica sinensis*, *Rehmannia glutinosa*, *Platycodon grandiflorum*, *Achyranthes bidentata* and *Citrus aurantium* from TCMHD, respectively. XFZY groups overlapped with SH formula with 23 compounds.

### Systematic docking

According to the information from previous studies or the ligand location in complex structure, we defined binding site for each protein, as shown in Table 2. For each binding site, systematic docking was performed by four docking software, including AutoDock, AutoDock Vina, Glide SP and XP.

#### AutoDock 4.2

Around each binding site, a grid box was created whose volume is large enough to encompass the entire binding site and accommodate the TCM molecules used for docking. Before molecular docking was performed, polar hydrogens and Gasteiger partial charges were added to proteins and ligands by AutoDockTools^27^. The conformations of proteins and ligands were set to rigid and flexible, respectively. Using AutoDock 4.2^27^, we conducted molecular docking by genetic algorithm and ranked the docking results by empirical free energy function.

#### AutoDock Vina

All the input files for AutoDock 4.2 docking were also used for the AutoDock Vina^28^ docking, including target protein structure, Gasteiger partial charges and the grid box dimensions. The remaining parameter was set to default.

#### Glide 6.9

All the modules used in Glide docking procedure is implemented by Schrödinger version 2015-4^42^. Each target protein is firstly subjected to protein preparation wizard to assign bond orders and add hydrogens. Meanwhile, all the compounds were treated by LigPrep module with default parameters. For the grid generation step, the binding site was defined by a rectangular box centered on the centroid of pocket residues with x, and z direction range from 10 to 20 Å according to pocket volume. Both the standard precision (SP)^29^ and extra precision (XP)^30^ methods are used for molecular docking and the scoring function is GlideScore. The ligand sampling method is flexible and no additional constraints were applied in Glide docking. Post docking minimizations were performed for each docking results.

#### Integration of docking results

Firstly, each interaction relationship between one compound and one protein was explored and scored by four docking tools independently. For certain target proteins, for example, gp120, compounds scored as top 40% by all four docking tools were regarded as potential inhibitors of gp120. Then, the docking poses of these potential inhibitors generated by each docking tool were compared and the RMSD between them were calculated. After that, we selected the compounds with similar docking pose (RMSD < 2 Å) by at least three docking tools. These top-ranked and RSMD-restricted compounds were regarded as anti-gp120 inhibitors, and their poses and scores generated by Glide XP were used for further study.

## Additional Information

**How to cite this article**: Liang, H. *et al*. Herb-target interaction network analysis helps to disclose molecular mechanism of traditional Chinese medicine. *Sci. Rep*. **6**, 36767; doi: 10.1038/srep36767 (2016).

**Publisher’s note:** Springer Nature remains neutral with regard to jurisdictional claims in published maps and institutional affiliations.

## Supplementary Material

Supplementary Information

## Figures and Tables

**Table 1:** Number of potential inhibitors in each group to 17 viral proteins.

	SH formula	AR group	SP group	XFZY group
PR	5	1	1	0
RT1	4	4	8	1
RT2	0	0	0	0
IN	7	0	5	2
NC	5	3	4	3
CA1	3	7	5	1
CA2	6	3	6	0
MA	0	0	1	0
Nef1	0	1	1	0
Nef2	1	0	1	0
Vpr	0	1	1	2
gp120	4	2	4	0
gp41	2	1	1	0
CCR5	3	1	2	0
CXCR4	4	0	1	0
CycT1	8	0	4	0
ELOC	5	1	7	0
Total	57	25	52	9

**Table 2:** Proteins, structures and binding sites used in molecular docking.

Viral Protein	Structure (PDB ID)	Binding Site
PR	2I4U^43^	Substrate binding site
RT1	3KK1^44^	Inhibitor GS-9148-diphosphate binding site
RT2	1VRT^45^	Inhibitor nevirapine binding site
IN	3LPU^46^	LEDGF/p75 binding site
NC	2M3Z^47^	Zinc knuckle
CA1	4NX4^48^	Inhibitor CAP-1 binding site
CA2	2XDE^49^	Inhibitor PF-3450074 binding site
MA	2GOL^50^	PI(4,5)P2 binding site^51^
Nef1	1AVZ^52^	SH3 binding site^53^
Nef2	1EFN^54^	Nef dimerization site^55^
Vpr	1M8L^56^	Inhibitor vipirinin binding site^57^
gp120	4DKR^58^	CD4 binding site
gp41	1AIK^59^	C34 WWI residues binding site^60^
**Host Protein**	**Structure (PDB ID)**	**Binding Site**
CCR5	4MBS^61^	Inhibitor maraviroc binding site
CXCR4	3ODU^62^	Antagonist IT1t binding site
CycT1	Homology modeling based on 2W2H^35^,^36^	Tat/TAR RNA recognition motif
ELOC	4N9F^63^	Vif binding site^64^
