# Supplementary material for: Herb-target interaction network analysis helps to disclose molecular mechanism of traditional Chinese medicine
Source: Sci Rep. 2016 Nov 11;6:36767. doi: 10.1038/srep36767 (PMC5105066; doi:10.1038/srep36767)
Supplement: Supplementary Information [file srep36767-s1.pdf]

Supplementary Information

Herb-target interaction network analysis helps to disclose molecular mechanism of traditional Chinese medicine

Hao Liang[1], Hao Ruan[2], Qi Ouyang[1,3,4] and Luhua Lai[1,2,3,4,*]

1 Peking-Tsinghua Center for Life Sciences, Academy for Advanced Interdisciplinary Studies, Peking University, Beijing 100871, China.

2 Beijing National Laboratory for Molecular Sciences, State Key Laboratory for Structural Chemistry of Unstable and Stable Species, College of Chemistry and Molecular Engineering, Peking University, Beijing 100871, China.

3 State Key Laboratory for Artificial Microstructures and Mesoscopic Physics, School of Physics, Peking University, Beijing 100871, China.

4 Center for Quantitative Biology, Academy for Advanced Interdisciplinary Studies, Peking University, Beijing 100871, China.

*To whom correspondence should be addressed.

Correspondence and requests for materials should be addressed to L.L. (lhlai@pku.edu.cn)

Contents

Supplementary Figure S1. Chemical property distributions of SH formula, AR group and SP group.
Supplementary Figure S2. Herb-target network of HIV-1 related proteins and SH formula, AR group, SP group and XFZY group (top 30% cutoff).
Supplementary Figure S3. Herb-target network of HIV-1 related proteins and SH formula, AR group, SP group and XFZY group (top 50% cutoff).
Supplementary Figure S4. Overlap compounds between SH formula and control groups.
Supplementary Table S1 HTF of active herbs in SH formula against 17 viral proteins
Supplementary Table S2 HTF of active herbs in AR group against 17 viral proteins
Supplementary Table S3 HTF of active herbs in SP group against 17 viral proteins
Supplementary Table S4 HTF of active herbs in XFZY group against 17 viral proteins
Supplementary Table S5 EFs of overall and individual herbs of SH formula and XFZY groups
Supplementary Table S6. Number of potential inhibitors in each group to 17 viral proteins (top 10% cutoff)
Supplementary Table S7. Number of potential inhibitors in each group to 17 viral proteins (top 20% cutoff)
Supplementary Table S8 Number of potential inhibitors in each group to 17 viral proteins (top 30% cutoff)
Supplementary Table S9 HTF of active herbs in SH group against 17 viral proteins (top 30% cutoff)
Supplementary Table S10 HTF of active herbs in AR group against 17 viral proteins (top 30% cutoff)
Supplementary Table S11 HTF of active herbs in SP group against 17 viral proteins (top 30% cutoff)
Supplementary Table S12 HTF of active herbs in XFZY group against 17 viral proteins (top 30% cutoff)
Supplementary Table S13 Number of potential inhibitors in each group to 17 viral proteins (top 50% cutoff)
Supplementary Table S14 HTF of active herbs in SH group against 17 viral proteins (top 50% cutoff)
Supplementary Table S15 HTF of active herbs in AR group against 17 viral proteins (top 50% cutoff)
Supplementary Table S16 HTF of active herbs in SP group against 17 viral proteins (top 50% cutoff)
Supplementary Table S17 HTF of active herbs in XFZY group against 17 viral proteins (top 50% cutoff)
Supplementary Table S18. The Latin name, Chinese name and compound number of TCMHD herbs.

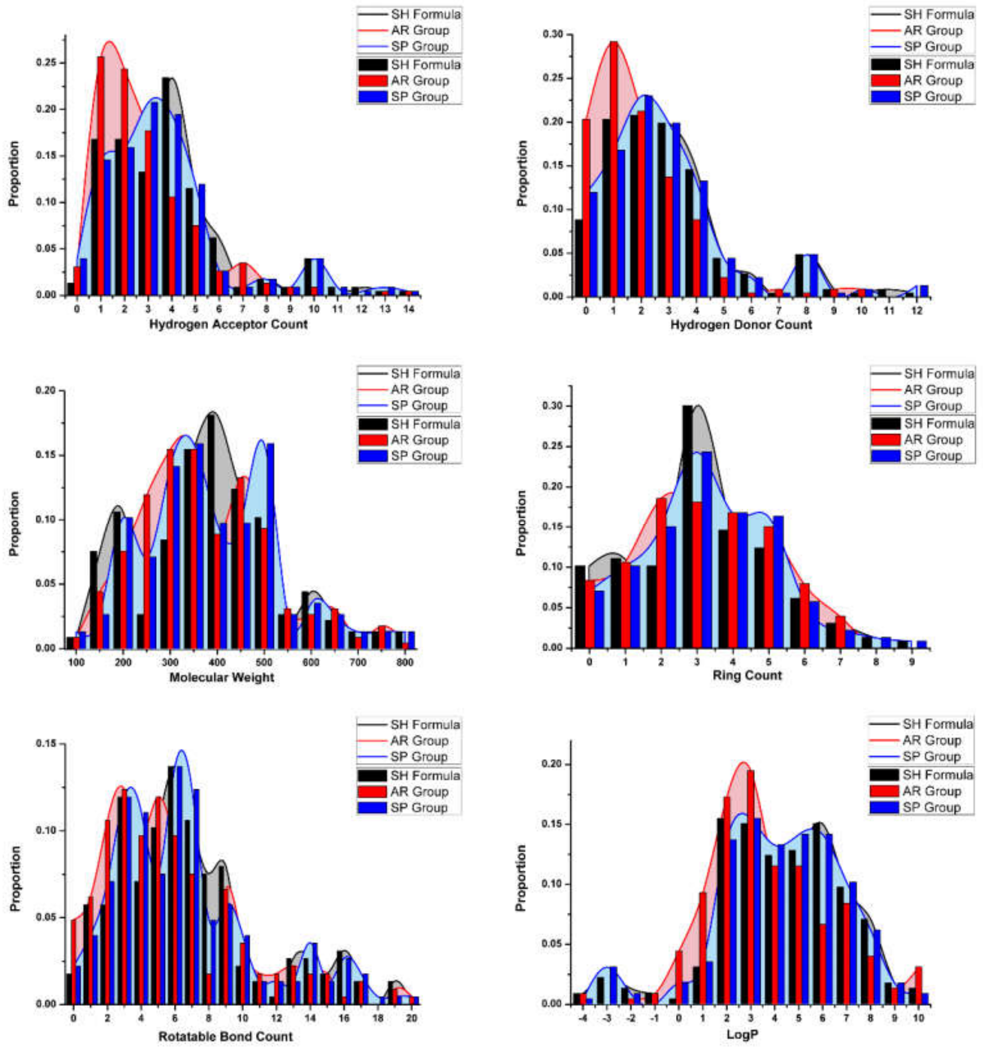

Supplementary Figure S1. Chemical property distributions of SH formula (black histogram and grey area), AR group (red histogram and pink area) and SP group (blue histogram and cyan area).

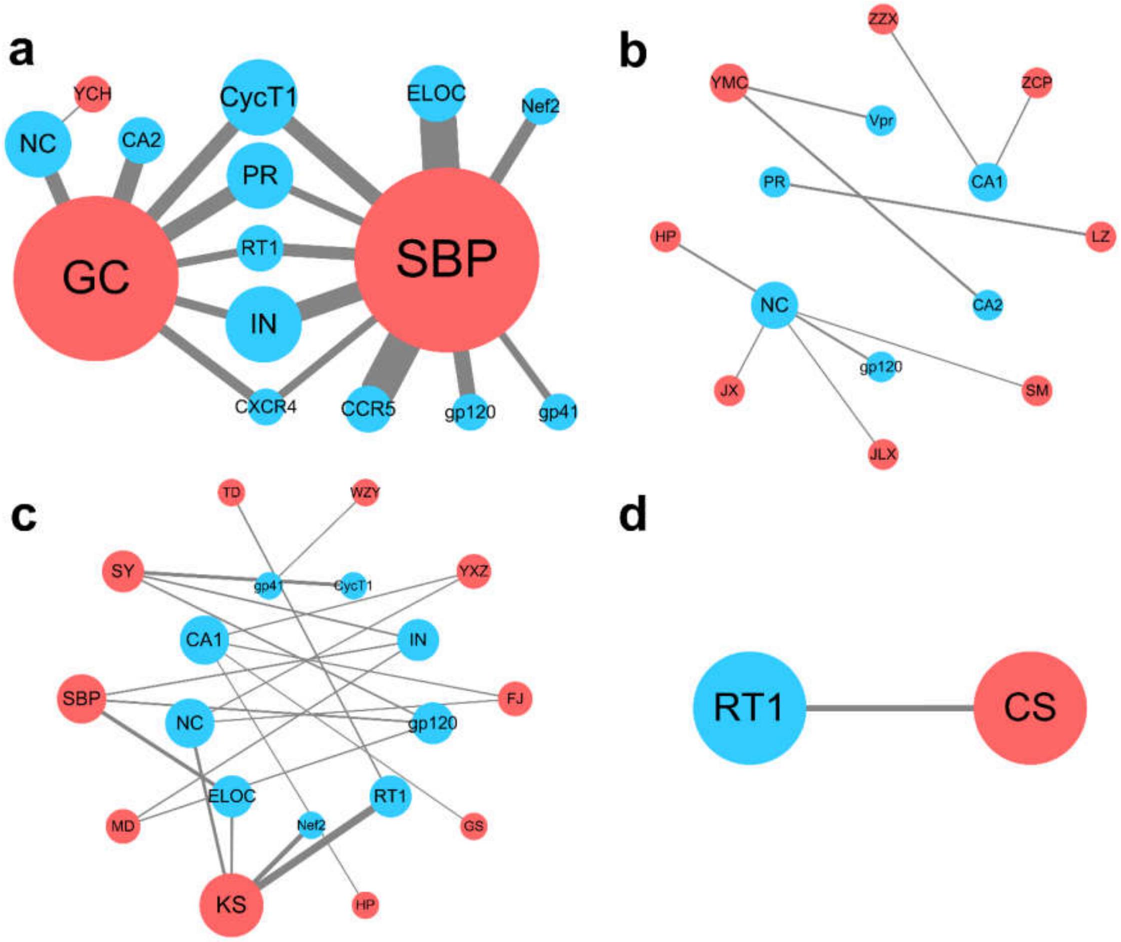

Supplementary Figure S2. Herb-target network of HIV-1 related proteins and SH formula (a), AR group (b), SP group (c) and XFZY group (d). The cutoff is set as top 30% by all four software. GC, SBP, YCH, and CS represents *Glycyrrhiza uralensis*, *Morus alba*, *Artemisia capillaries* and *Paeonia rubra*, respectively. Node sizes of herbs and targets are weighted by active compound numbers, edge sizes are weighted by HTFs.

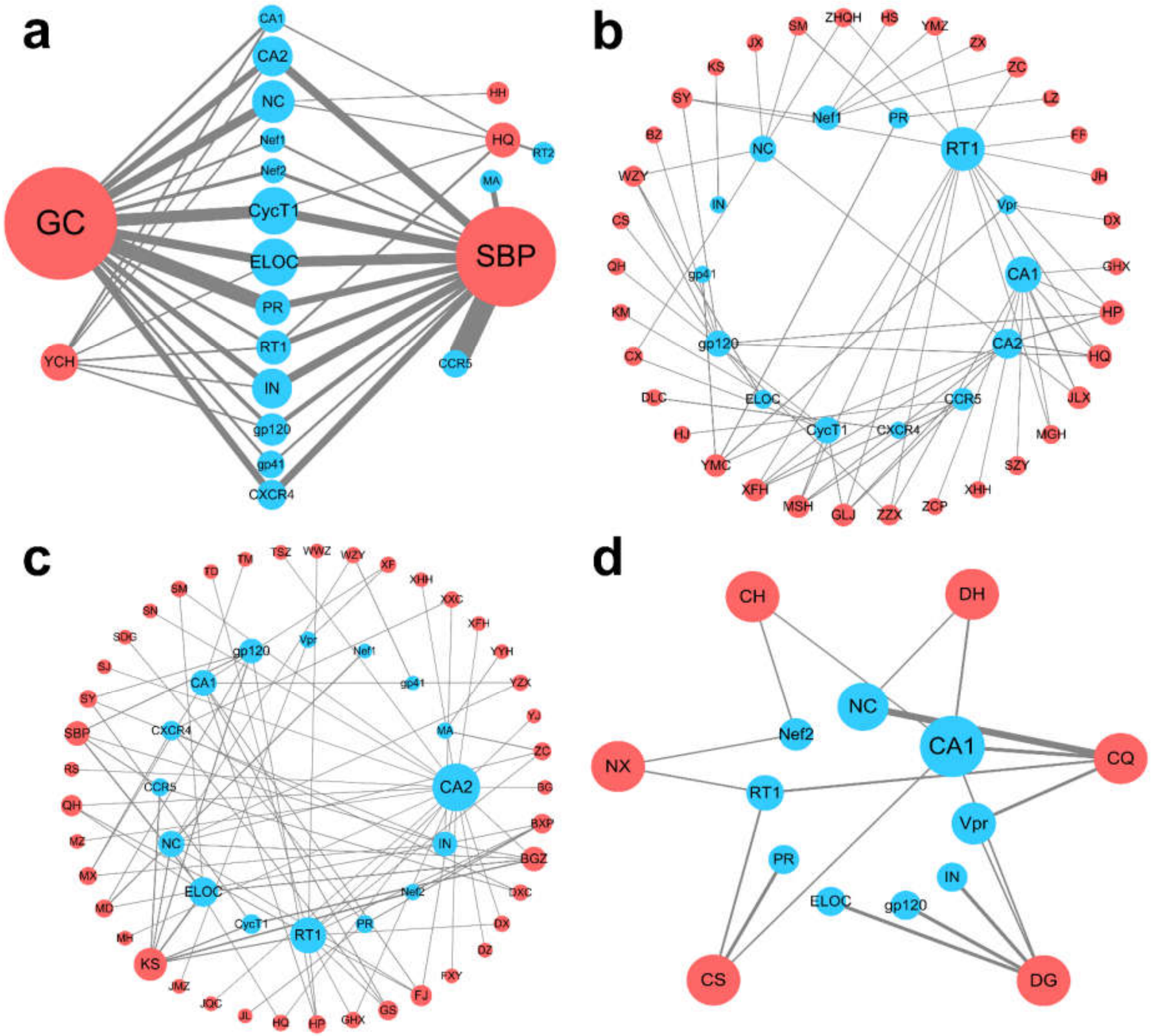

Supplementary Figure S3. Herb-target network of HIV-1 related proteins and SH formula (a), AR group (b), SP group (c) and XFZY group (d). The cutoff is set as top 50% by all four software. GC, HH, HQ, SBP, YCH, CH, CQ, CS, DG, DH, NX represents *Glycyrrhiza uralensis*, *Carthamus tinctorius*, *Astragalus membranaceus*, *Morus alba*, *Artemisia*

capillaries, *Bupleurum chinense*, *Ligusticum wallichii*, *Paeonia rubra*, *Angelica sinensis*, *Rehmannia glutinosa* and *Achyranthes bidentata* respectively. Node sizes of herbs and targets are weighted by active compound numbers, edge sizes are weighted by HTFs.

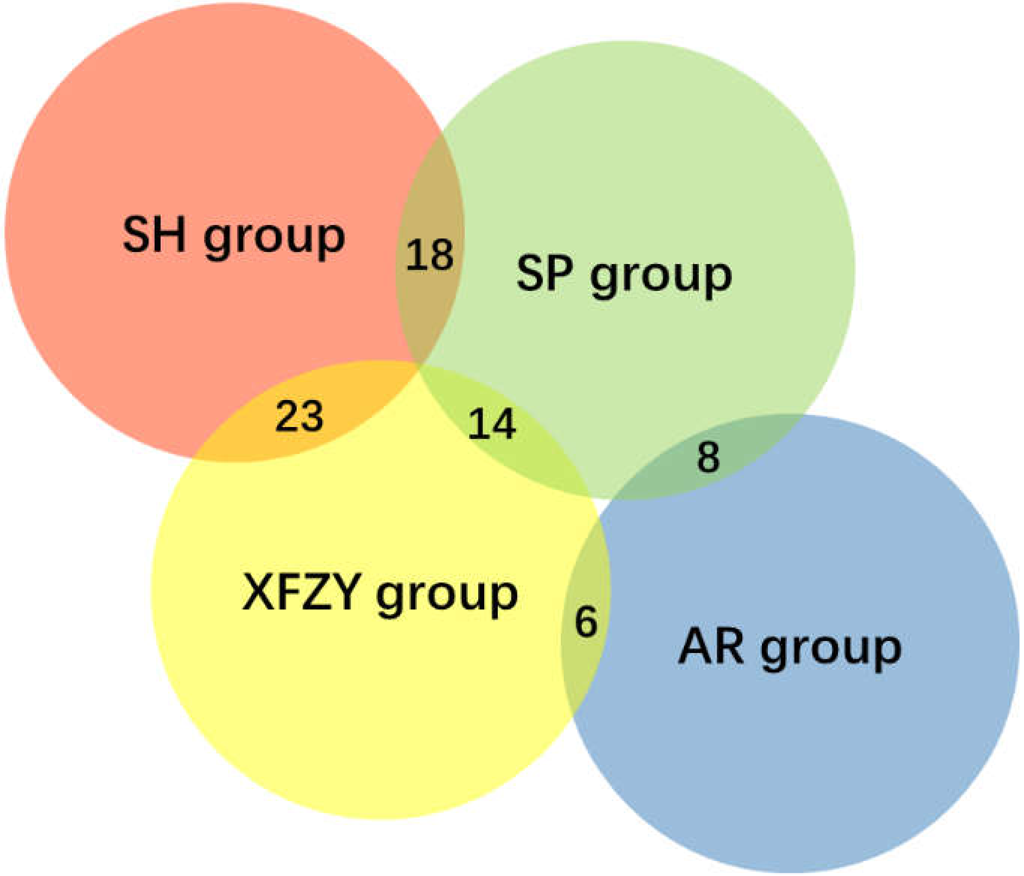

Supplementary Figure S4. Overlap compounds between SH formula and control groups.

Supplementary Table S1. HTF of active herbs in SH formula against 17 viral proteins.

Herb	PR	RT1	RT2	IN	NC	CA1	CA2	MA	Nef1	Nef2	Vpr	gp120	gp41	CCR5	CXCR4	CycT1	ELOC
Glycyrrhiza uralensis	107.65	33.83	0.00	83.80	62.67	24.70	41.27	0.00	0.00	0.00	0.00	16.60	11.85	0.00	65.20	79.03	0.00
Carthamus tinctorius	0.00	0.00	0.00	0.00	1.46	0.00	0.00	0.00	0.00	0.00	0.00	0.00	0.00	0.00	0.00	0.00	0.00
Astragalus membranaceus	0.00	8.62	0.00	0.00	0.00	5.92	0.00	0.00	0.00	0.00	0.00	0.00	0.00	0.00	0.00	5.21	0.00
Morus alba	75.02	78.98	0.00	133.87	0.00	0.00	69.70	0.00	0.00	61.82	0.00	44.15	17.66	248.05	75.35	77.22	136.78
Artemisia capillaries	0.00	0.00	0.00	0.00	10.10	13.35	9.08	0.00	0.00	0.00	0.00	8.57	0.00	0.00	0.00	0.00	11.85

Supplementary Table S2. HTF of active herbs in AR group against 17 viral proteins.

Herb	Abbr.	PR	RT1	RT2	IN	NC	CA1	CA2	MA	Nef1	Nef2	Vpr	gp120	gp41	CCR5	CXCR4	CycT1	ELOC
Magnolia officinalis	HP	0.00	11.13	0.00	0.00	0.00	0.00	7.75	0.00	0.00	0.00	0.00	10.62	0.00	0.00	0.00	0.00	0.00
Scutellaria baicalensis	HQN	0.00	0.00	0.00	0.00	0.00	2.27	3.98	0.00	0.00	0.00	0.00	0.00	0.00	0.00	0.00	0.00	0.00
Piper nigrum	HJ	0.00	0.00	0.00	0.00	0.00	0.00	0.00	0.00	0.00	0.00	0.00	0.00	0.00	6.90	0.00	0.00	0.00
Dalbergia odorifera	JX	0.00	0.00	0.00	0.00	2.20	0.00	0.00	0.00	0.00	0.00	0.00	0.00	0.00	0.00	0.00	0.00	0.00
Murraya paniculata	JLX	0.00	8.64	0.00	0.00	5.54	2.60	0.00	0.00	0.00	0.00	0.00	0.00	0.00	0.00	0.00	0.00	0.00
Ganoderma lucidum	LZ	8.95	0.00	0.00	0.00	0.00	0.00	0.00	0.00	0.00	0.00	0.00	0.00	0.00	0.00	0.00	0.00	0.00
Rosa rugosa	MGH	0.00	0.00	0.00	0.00	0.00	0.98	0.00	0.00	0.00	0.00	0.00	0.00	0.00	0.00	0.00	0.00	0.00
Cornus officinalis	SZY	0.00	0.00	0.00	0.00	0.00	1.04	0.00	0.00	0.00	0.00	0.00	0.00	0.00	0.00	0.00	0.00	0.00
Caesalpinia sappan	SM	0.00	0.00	0.00	0.00	2.20	0.00	0.00	0.00	0.00	0.00	0.00	0.00	0.00	0.00	0.00	0.00	0.00
Evodia rutaecarpa	WZY	0.00	0.00	0.00	0.00	0.00	0.00	0.00	0.00	0.00	0.00	0.00	7.53	0.00	0.00	0.00	0.00	9.06
Crocus sativus	XHH	0.00	0.00	0.00	0.00	0.00	1.05	0.00	0.00	0.00	0.00	0.00	0.00	0.00	0.00	0.00	0.00	0.00
Leonurus artemisia	YMC	0.00	0.00	0.00	0.00	0.00	0.00	4.57	0.00	0.00	0.00	11.76	0.00	5.22	0.00	0.00	0.00	0.00
Acorus calamus	ZCP	0.00	0.00	0.00	0.00	0.00	0.90	0.00	0.00	0.00	0.00	0.00	0.00	0.00	0.00	0.00	0.00	0.00
Alisma orientale	ZX	0.00	0.00	0.00	0.00	0.00	0.00	0.00	0.00	3.82	0.00	0.00	0.00	0.00	0.00	0.00	0.00	0.00
Valeriana jatamansii	ZZX	0.00	0.00	0.00	0.00	0.00	1.25	0.00	0.00	0.00	0.00	0.00	0.00	0.00	0.00	0.00	0.00	0.00
Arnebia euchroma	ZC	0.00	7.04	0.00	0.00	0.00	0.00	0.00	0.00	0.00	0.00	0.00	0.00	0.00	0.00	0.00	0.00	0.00

Supplementary Table S3. HTF of active herbs in SP group against 17 viral proteins.

Herb	Abbr.	PR	RT1	RT2	IN	NC	CA1	CA2	MA	Nef1	Nef2	Vpr	gp120	gp41	CCR5	CXCR4	CycT1	ELOC
Dictamnus dasycarpus	BXP	17.61	3.83	0.00	2.95	0.00	0.00	0.00	0.00	0.00	0.00	0.00	0.00	0.00	0.00	0.00	0.00	0.00
Psoralea corylifolia	BGZ	0.00	0.00	0.00	4.14	0.00	0.00	3.03	0.00	0.00	0.00	0.00	0.00	0.00	0.00	0.00	14.89	13.93
Juncus effuses	DXC	0.00	0.00	0.00	0.00	0.00	0.00	0.00	0.00	0.00	0.00	0.00	0.00	0.00	0.00	6.28	0.00	0.00
Eugenia caryophyllata	DX	0.00	0.00	0.00	0.00	0.00	0.00	1.06	0.00	0.00	0.00	0.00	0.00	0.00	0.00	0.00	0.00	0.00
Apis mellifera	FJ	0.00	0.00	0.00	0.00	5.20	4.62	3.19	0.00	0.00	0.00	0.00	0.00	0.00	0.00	0.00	0.00	0.00
Nardostachys chinensis	GS	0.00	2.44	0.00	0.00	0.00	2.94	0.00	0.00	0.00	0.00	0.00	0.00	0.00	0.00	0.00	0.00	0.00
Pogostemon cablin	GHX	0.00	0.00	0.00	0.00	0.00	0.00	1.06	0.00	0.00	0.00	0.00	0.00	0.00	0.00	0.00	0.00	0.00
Magnolia officinalis	HP	0.00	3.10	0.00	0.00	0.00	3.30	0.00	0.00	0.00	0.00	0.00	0.00	0.00	0.00	0.00	0.00	0.00
Cassia tora	JMZ	0.00	0.00	0.00	0.00	0.00	0.00	0.00	0.00	0.00	0.00	2.66	0.00	0.00	0.00	0.00	0.00	0.00
Sophora flavescens	KS	0.00	19.84	0.00	0.00	21.32	0.00	0.00	0.00	0.00	39.84	0.00	0.00	0.00	17.04	0.00	12.18	20.08
Ophiopogon japonicus	MD	0.00	0.00	0.00	4.04	0.00	0.00	0.00	0.00	11.40	0.00	0.00	6.84	0.00	0.00	0.00	0.00	0.00
Artemisia annua	QH	0.00	2.94	0.00	0.00	0.00	0.00	2.13	0.00	0.00	0.00	0.00	0.00	0.00	0.00	0.00	0.00	0.00
Morus alba	SBP	0.00	0.00	0.00	4.23	0.00	0.00	0.00	0.00	0.00	0.00	0.00	16.05	0.00	11.70	0.00	0.00	26.76
Morus alba	SY	0.00	0.00	0.00	3.91	0.00	0.00	0.00	0.00	0.00	0.00	0.00	6.20	0.00	0.00	0.00	5.85	0.00
Caesalpinia sappan	SM	0.00	0.00	0.00	0.00	0.00	0.00	1.09	0.00	0.00	0.00	0.00	0.00	0.00	0.00	0.00	0.00	0.00
Asparagus cochinchinensis	TD	0.00	1.57	0.00	0.00	0.00	0.00	0.00	0.00	0.00	0.00	0.00	0.00	0.00	0.00	0.00	0.00	0.00

Supplementary Table S4. HTF of active herbs in XFZY group against 17 viral proteins.

Herb	PR	RT1	RT2	IN	NC	CA1	CA2	MA	Nef1	Nef2	Vpr	gp120	gp41	CCR5	CXCR4	CycT1	ELOC
Ligusticum wallichii	0.00	0.00	0.00	10.02	56.24	39.32	0.00	0.00	0.00	0.00	22.12	0.00	0.00	0.00	0.00	0.00	0.00
Paeonia rubra	0.00	10.53	0.00	0.00	0.00	0.00	0.00	0.00	0.00	0.00	0.00	0.00	0.00	0.00	0.00	0.00	0.00
Angelica sinensis	0.00	0.00	0.00	2.50	0.00	0.00	0.00	0.00	0.00	0.00	0.00	0.00	0.00	0.00	0.00	0.00	0.00

Supplementary Table S5. EFs of overall and individual herbs of SH formula and XFZY groups.

Herbs	PR	RT1	RT2	IN	NC	CA1	CA2	MA	Nef1	Nef2	Vpr	gp120	gp41	CCR5	CXCR4	CycT1	ELOC
**SH formula**	1.64	0.57	0.00	5.24	1.00	0.60	6.36	0.00	0.00	0.34	0.00	1.34	1.21	1.24	1.89	4.66	2.46
Glycyrrhiza uralensis	2.64	0.38	0.00	6.05	1.61	0.54	5.71	0.00	0.00	0.00	0.00	0.90	1.62	0.00	2.54	6.27	0.00
Carthamus tinctorius	0.00	0.00	0.00	0.00	1.41	0.00	0.00	0.00	0.00	0.00	0.00	0.00	0.00	0.00	0.00	0.00	0.00
Astragalus membranaceus	0.00	2.01	0.00	0.00	0.00	2.85	0.00	0.00	0.00	0.00	0.00	0.00	0.00	0.00	0.00	8.23	0.00
Morus alba	3.15	1.37	0.00	14.41	0.00	0.00	15.29	0.00	0.00	1.62	0.00	3.21	2.90	5.95	4.54	8.40	9.46
Artemisia capillaries	0.00	0.00	0.00	0.00	0.83	0.84	4.44	0.00	0.00	0.00	0.00	1.40	0.00	0.00	0.00	0.00	2.06
**XFZY formula**	0.00	0.14	0.00	1.48	0.59	0.20	0.00	0.00	0.00	0.00	0.82	0.00	0.00	0.00	0.00	0.00	0.00
Bupleurum chinense	0.00	0.00	0.00	0.00	0.00	0.00	0.00	0.00	0.00	0.00	0.00	0.00	0.00	0.00	0.00	0.00	0.00
Ligusticum wallichii	0.00	0.00	0.00	2.92	2.33	0.78	0.00	0.00	0.00	0.00	3.24	0.00	0.00	0.00	0.00	0.00	0.00
Paeonia rubra	0.00	3.58	0.00	0.00	0.00	0.00	0.00	0.00	0.00	0.00	0.00	0.00	0.00	0.00	0.00	0.00	0.00
Angelica sinensis	0.00	0.00	0.00	3.32	0.00	0.00	0.00	0.00	0.00	0.00	0.00	0.00	0.00	0.00	0.00	0.00	0.00
Rehmannia glutinosa	0.00	0.00	0.00	0.00	0.00	0.00	0.00	0.00	0.00	0.00	0.00	0.00	0.00	0.00	0.00	0.00	0.00
Platycodon grandiflorum	0.00	0.00	0.00	0.00	0.00	0.00	0.00	0.00	0.00	0.00	0.00	0.00	0.00	0.00	0.00	0.00	0.00
Achyranthes bidentata	0.00	0.00	0.00	0.00	0.00	0.00	0.00	0.00	0.00	0.00	0.00	0.00	0.00	0.00	0.00	0.00	0.00

Supplementary Table S6. Number of potential inhibitors in each group to 17 viral proteins (top 10% cutoff).

Group	PR	RT1	RT2	IN	NC	CA1	CA2	MA	Nef1	Nef2	Vpr	gp120	gp41	CCR5	CXCR4	CycT1	ELOC	Total
**SH formula**	0	0	0	0	0	0	0	0	0	0	0	0	0	0	0	0	0	0
**AR group**	0	0	0	0	0	1	0	0	0	0	0	0	0	0	0	0	0	1
**SP group**	0	1	0	0	0	0	0	0	0	0	0	0	0	0	0	0	0	1
**XFZY group**	0	0	0	0	0	0	0	0	0	0	0	0	0	0	0	0	0	0

Supplementary Table S7. Number of potential inhibitors in each group to 17 viral proteins (top 20% cutoff).

Group	PR	RT1	RT2	IN	NC	CA1	CA2	MA	Nef1	Nef2	Vpr	gp120	gp41	CCR5	CXCR4	CycT1	ELOC	Total
**SH formula**	0	2	0	1	2	0	1	0	0	0	0	1	0	1	1	1	1	11
**AR group**	1	0	0	0	0	1	0	0	0	0	1	0	0	0	0	0	0	3
**SP group**	0	2	0	0	0	0	0	0	0	0	0	2	1	0	0	0	2	7
**XFZY group**	0	0	0	0	0	0	0	0	0	0	0	0	0	0	0	0	0	0

Supplementary Table S8. Number of potential inhibitors in each group to 17 viral proteins (top 30% cutoff).

Group	PR	RT1	RT2	IN	NC	CA1	CA2	MA	Nef1	Nef2	Vpr	gp120	gp41	CCR5	CXCR4	CycT1	ELOC	Total
**SH formula**	4	2	0	5	4	0	2	0	0	1	0	1	1	2	1	5	3	31
**AR group**	1	0	0	0	3	2	1	0	0	0	1	1	0	0	0	0	0	9
**SP group**	0	3	0	3	4	4	0	0	0	1	0	3	1	0	0	1	3	23
**XFZY group**	0	1	0	0	0	0	0	0	0	0	0	0	0	0	0	0	0	1

Supplementary Table S9. HTF of active herbs in SH group against 17 viral proteins (top 30% cutoff).

Herb	PR	RT1	RT2	IN	NC	CA1	CA2	MA	Nef1	Nef2	Vpr	gp120	gp41	CCR5	CXCR4	CycT1	ELOC
Glycyrrhiza uralensis	75.36	35.52	0.00	40.32	65.80	0.00	86.66	0.00	0.00	0.00	0.00	0.00	0.00	0.00	45.64	64.30	0.00
Morus alba	37.05	56.20	0.00	90.45	0.00	0.00	0.00	0.00	0.00	56.20	0.00	72.60	32.10	167.00	35.20	71.45	175.60
Artemisia capillaries	0.00	0.00	0.00	0.00	3.03	0.00	0.00	0.00	0.00	0.00	0.00	0.00	0.00	0.00	0.00	0.00	0.00

Supplementary Table S10. HTF of active herbs in AR group against 17 viral proteins (top 30% cutoff).

Herb	Abbr.	PR	RT1	RT2	IN	NC	CA1	CA2	MA	Nef1	Nef2	Vpr	gp120	gp41	CCR5	CXCR4	CycT1	ELOC
Magnolia officinalis	HP	0.00	0.00	0.00	0.00	0.00	0.00	0.00	0.00	0.00	0.00	0.00	7.08	0.00	0.00	0.00	0.00	0.00
Dalbergia odorifera	JX	0.00	0.00	0.00	0.00	2.20	0.00	0.00	0.00	0.00	0.00	0.00	0.00	0.00	0.00	0.00	0.00	0.00
Murraya paniculata	JLX	0.00	0.00	0.00	0.00	1.85	0.00	0.00	0.00	0.00	0.00	0.00	0.00	0.00	0.00	0.00	0.00	0.00
Ganoderma lucidum	LZ	8.95	0.00	0.00	0.00	0.00	0.00	0.00	0.00	0.00	0.00	0.00	0.00	0.00	0.00	0.00	0.00	0.00
Caesalpinia sappan	SM	0.00	0.00	0.00	0.00	2.20	0.00	0.00	0.00	0.00	0.00	0.00	0.00	0.00	0.00	0.00	0.00	0.00
Leonurus artemisia	YMC	0.00	0.00	0.00	0.00	0.00	0.00	9.14	0.00	0.00	0.00	7.84	0.00	0.00	0.00	0.00	0.00	0.00
Acorus calamus	ZCP	0.00	0.00	0.00	0.00	0.00	3.14	0.00	0.00	0.00	0.00	0.00	0.00	0.00	0.00	0.00	0.00	0.00
Valeriana jatamansii	ZZX	0.00	0.00	0.00	0.00	0.00	4.38	0.00	0.00	0.00	0.00	0.00	0.00	0.00	0.00	0.00	0.00	0.00

Supplementary Table S11. HTF of active herbs in SP group against 17 viral proteins (top 30% cutoff).

Herb	Abbr.	PR	RT1	RT2	IN	NC	CA1	CA2	MA	Nef1	Nef2	Vpr	gp120	gp41	CCR5	CXCR4	CycT1	ELOC
Apis mellifera	FJ	0.00	0.00	0.00	0.00	3.47	3.85	0.00	0.00	0.00	0.00	0.00	0.00	0.00	0.00	0.00	0.00	0.00
Nardostachys chinensis	GS	0.00	0.00	0.00	0.00	0.00	1.83	0.00	0.00	0.00	0.00	0.00	0.00	0.00	0.00	0.00	0.00	0.00
Magnolia officinalis	HP	0.00	0.00	0.00	0.00	0.00	2.06	0.00	0.00	0.00	0.00	0.00	0.00	0.00	0.00	0.00	0.00	0.00
Sophora flavescens	KS	0.00	46.30	0.00	0.00	14.21	0.00	0.00	0.00	0.00	26.56	0.00	0.00	0.00	0.00	0.00	0.00	10.42
Ophiopogon japonicus	MD	0.00	0.00	0.00	4.49	0.00	0.00	0.00	0.00	0.00	0.00	0.00	4.56	0.00	0.00	0.00	0.00	0.00
Morus alba	SBP	0.00	0.00	0.00	5.29	0.00	0.00	0.00	0.00	0.00	0.00	0.00	7.26	0.00	0.00	0.00	0.00	19.14
Morus alba	SY	0.00	0.00	0.00	6.51	0.00	0.00	0.00	0.00	0.00	0.00	0.00	6.20	0.00	0.00	0.00	17.55	0.00
Asparagus cochinchinensis	TD	0.00	5.49	0.00	0.00	0.00	0.00	0.00	0.00	0.00	0.00	0.00	0.00	0.00	0.00	0.00	0.00	0.00
Evodia rutaecarpa	WZY	0.00	0.00	0.00	0.00	0.00	0.00	0.00	0.00	0.00	0.00	0.00	0.00	2.66	0.00	0.00	0.00	0.00
Alpinia oxyphylla	YZX	0.00	0.00	0.00	0.00	3.47	3.85	0.00	0.00	0.00	0.00	0.00	0.00	0.00	0.00	0.00	0.00	0.00

Supplementary Table S12. HTF of active herbs in XFZY group against 17 viral proteins (top 30% cutoff).

Herb	PR	RT1	RT2	IN	NC	CA1	CA2	MA	Nef1	Nef2	Vpr	gp120	gp41	CCR5	CXCR4	CycT1	ELOC
Paeonia rubra	0.00	10.53	0.00	0.00	0.00	0.00	0.00	0.00	0.00	0.00	0.00	0.00	0.00	0.00	0.00	0.00	0.00

Supplementary Table S13. Number of potential inhibitors in each group to 17 viral proteins (top 50% cutoff).

Group	PR	RT1	RT2	IN	NC	CA1	CA2	MA	Nef1	Nef2	Vpr	gp120	gp41	CCR5	CXCR4	CycT1	ELOC	Total
**SH formula**	6	6	1	8	9	3	8	1	2	2	0	5	3	3	4	11	11	83
**AR group**	3	15	0	1	6	11	8	0	5	0	2	6	1	4	1	6	2	71
**SP group**	3	14	0	7	8	8	21	2	1	2	2	7	1	3	4	4	10	97
**XFZY group**	1	3	0	1	7	11	0	0	0	2	5	1	0	0	0	0	1	32

Supplementary Table S14. HTF of active herbs in SH group against 17 viral proteins (top 50% cutoff).

Herb	PR	RT1	RT2	IN	NC	CA1	CA2	MA	Nef1	Nef2	Vpr	gp120	gp41	CCR5	CXCR4	CycT1	ELOC
Glycyrrhiza uralensis	193.70	32.99	0.00	72.63	115.18	32.11	77.65	0.00	20.34	37.38	0.00	42.42	23.34	0.00	84.76	153.75	111.89
Carthamus tinctorius	0.00	0.00	0.00	0.00	1.09	0.00	0.00	0.00	0.00	0.00	0.00	0.00	0.00	0.00	0.00	0.00	0.00
Astragalus membranaceus	0.00	21.36	22.10	0.00	6.41	9.87	0.00	0.00	0.00	0.00	0.00	0.00	0.00	0.00	0.00	8.68	0.00
Morus alba	88.66	70.00	0.00	105.47	0.00	0.00	105.69	62.79	19.63	36.53	0.00	52.17	20.86	293.15	89.05	112.62	127.62
Artemisia capillaries	0.00	14.46	0.00	12.58	10.60	18.69	12.72	0.00	0.00	0.00	0.00	11.99	0.00	0.00	0.00	0.00	11.06

Supplementary Table S15. HTF of active herbs in AR group against 17 viral proteins (top 50% cutoff).

Herb	Abbr.	PR	RT1	RT2	IN	NC	CA1	CA2	MA	Nef1	Nef2	Vpr	gp120	gp41	CCR5	CXCR4	CycT1	ELOC
Angelica dahurica	BZ	0.00	0.00	0.00	0.00	0.00	0.00	0.00	0.00	0.00	0.00	0.00	0.75	0.00	0.00	0.00	0.00	0.00
Aquilaria sinensis	CX	0.00	0.00	0.00	0.00	1.28	0.00	0.00	0.00	0.00	0.00	0.00	0.00	0.00	0.00	0.00	1.75	0.00
Eugenia caryophyllata	DX	0.00	0.00	0.00	0.00	0.00	0.00	0.00	0.00	0.00	0.00	1.45	0.00	0.00	0.00	0.00	0.00	0.00
Rabdosia rubescens	DLC	0.00	0.00	0.00	0.00	0.00	0.00	0.00	0.00	0.00	0.00	0.00	0.00	0.00	0.00	6.54	0.00	0.00
Saposhnikovia divaricata	FF	0.00	0.67	0.00	0.00	0.00	0.00	0.00	0.00	0.00	0.00	0.00	0.00	0.00	0.00	0.00	0.00	0.00
Alpinia officinarum	GLJ	0.00	2.55	0.00	0.00	0.00	0.00	4.45	0.00	0.00	0.00	0.00	0.00	0.00	6.59	0.00	4.54	0.00
Pogostemon cablin	GHX	0.00	0.00	0.00	0.00	0.00	0.64	0.00	0.00	0.00	0.00	0.00	0.00	0.00	0.00	0.00	0.00	0.00
Carpesium abrotanoides	HS	0.00	0.00	0.00	0.00	0.00	0.00	0.00	0.00	0.61	0.00	0.00	0.00	0.00	0.00	0.00	0.00	0.00
Magnolia officinalis	HP	0.00	3.18	0.00	0.00	0.00	3.52	9.53	0.00	0.00	0.00	0.00	4.72	0.00	0.00	0.00	0.00	0.00
Scutellaria baicalensis	HQN	0.00	3.19	0.00	0.00	0.00	3.54	8.42	0.00	0.00	0.00	0.00	4.51	0.00	0.00	0.00	0.00	0.00
Piper nigrum	HJ	0.00	0.00	0.00	0.00	0.00	0.00	0.00	0.00	0.00	0.00	0.00	0.00	0.00	1.73	0.00	0.00	0.00
Dalbergia odorifera	JX	0.00	0.00	0.00	0.00	1.10	0.00	0.00	0.00	0.00	0.00	0.00	0.00	0.00	0.00	0.00	0.00	0.00
Murraya paniculata	JLX	0.00	1.85	0.00	0.00	2.77	3.80	0.00	0.00	0.00	0.00	0.00	0.00	0.00	0.00	0.00	0.00	0.00
Chrysanthemum morifolium	JH	0.00	0.76	0.00	0.00	0.00	0.00	0.00	0.00	0.00	0.00	0.00	0.00	0.00	0.00	0.00	0.00	0.00
Picrasma quassioides	KM	0.00	0.00	0.00	0.00	0.00	0.00	0.00	0.00	0.00	0.00	0.00	0.00	0.00	0.00	0.00	1.14	0.00
Sophora flavescens	KS	0.00	0.00	0.00	5.28	0.00	0.00	0.00	0.00	0.00	0.00	0.00	0.00	0.00	0.00	0.00	0.00	0.00
Ganoderma lucidum	LZ	2.98	0.00	0.00	0.00	0.00	0.00	0.00	0.00	0.00	0.00	0.00	0.00	0.00	0.00	0.00	0.00	0.00
Rhododendron dauricum	MSH	0.00	2.55	0.00	0.00	0.00	0.00	4.45	0.00	0.00	0.00	0.00	0.00	0.00	6.59	0.00	4.54	0.00
Rosa rugosa	MGH	0.00	1.36	0.00	0.00	0.00	1.53	0.00	0.00	0.00	0.00	0.00	0.00	0.00	0.00	0.00	0.00	0.00
Artemisia annua	QH	0.00	0.00	0.00	0.00	0.00	0.00	0.00	0.00	0.00	0.00	0.00	0.00	0.00	0.00	0.00	0.73	0.00
Dioscorea opposita	SY	0.00	1.91	0.00	0.00	0.00	0.00	0.00	0.00	2.06	0.00	0.00	3.79	0.00	0.00	0.00	0.00	0.00
Cornus officinalis	SZY	0.00	0.00	0.00	0.00	0.00	1.37	0.00	0.00	0.00	0.00	0.00	0.00	0.00	0.00	0.00	0.00	0.00
Caesalpinia sappan	SM	4.79	0.00	0.00	0.00	2.20	0.00	0.00	0.00	0.00	0.00	0.00	0.00	0.00	0.00	0.00	0.00	0.00
Evodia rutaecarpa	WZY	0.00	0.00	0.00	0.00	1.88	0.00	0.00	0.00	0.00	0.00	0.00	3.77	0.00	0.00	0.00	0.00	6.79
Crocus sativus	XHH	0.00	0.00	0.00	0.00	0.00	0.81	0.00	0.00	0.00	0.00	0.00	0.00	0.00	0.00	0.00	0.00	0.00

Inula britannica	XFH	0.00	2.55	0.00	0.00	0.00	0.00	4.45	0.00	0.00	0.00	0.00	0.00	0.00	6.59	0.00	4.54	0.00
Eupatorium lindleyanum	YMZ	0.00	1.31	0.00	0.00	0.00	0.00	0.00	0.00	1.43	0.00	0.00	0.00	0.00	0.00	0.00	0.00	0.00
Leonurus artemisia	YMC	7.35	0.00	0.00	0.00	0.00	0.00	3.05	0.00	0.00	0.00	7.84	0.00	6.96	0.00	0.00	0.00	0.00
Acorus calamus	ZCP	0.00	0.00	0.00	0.00	0.00	0.70	0.00	0.00	0.00	0.00	0.00	0.00	0.00	0.00	0.00	0.00	0.00
Alisma orientale	ZX	0.00	0.00	0.00	0.00	0.00	0.00	0.00	0.00	0.76	0.00	0.00	0.00	0.00	0.00	0.00	0.00	0.00
Valeriana jatamansii	ZZX	0.00	1.72	0.00	0.00	0.00	2.92	0.00	0.00	0.00	0.00	0.00	2.12	0.00	0.00	0.00	0.00	0.00
Arnebia euchroma	ZC	0.00	3.02	0.00	0.00	0.00	0.00	0.00	0.00	1.32	0.00	0.00	0.00	0.00	0.00	0.00	0.00	0.00
Peucedanum decursivum	ZHQH	0.00	1.34	0.00	0.00	2.02	0.00	0.00	0.00	0.00	0.00	0.00	0.00	0.00	0.00	0.00	0.00	0.00

Supplementary Table S16. HTF of active herbs in SP group against 17 viral proteins (top 50% cutoff).

Herb	Abbr.	PR	RT1	RT2	IN	NC	CA1	CA2	MA	Nef1	Nef2	Vpr	gp120	gp41	CCR5	CXCR4	CycT1	ELOC
Ginkgo biloba	BG	0.00	0.00	0.00	0.00	0.00	0.00	0.35	0.00	0.00	0.00	0.00	0.00	0.00	0.00	0.00	0.00	0.00
Dictamnus dasycarpus	BXP	7.83	2.98	0.00	2.81	0.00	0.00	0.00	0.00	0.00	8.14	0.00	0.00	0.00	0.00	0.00	0.00	0.00
Psoralea corylifolia	BGZ	0.00	0.00	0.00	3.70	4.19	0.00	1.19	0.00	0.00	0.00	0.00	0.00	0.00	0.00	0.00	18.62	10.45
Juncus effuses	DXC	0.00	0.00	0.00	0.00	0.00	0.00	0.00	0.00	0.00	0.00	0.00	0.00	0.00	3.95	3.14	0.00	0.00
Eugenia caryophyllata	DX	0.00	1.57	0.00	0.00	0.00	0.00	0.67	0.00	0.00	0.00	0.00	0.00	0.00	0.00	0.00	0.00	0.00
Eucommia ulmoides	DZ	0.00	0.00	0.00	0.00	0.00	0.00	0.35	0.00	0.00	0.00	0.00	0.00	0.00	0.00	0.00	0.00	0.00
Cassia acutifolia	FXY	0.00	0.00	0.00	0.00	0.00	0.00	0.35	0.00	0.00	0.00	0.00	0.00	0.00	0.00	0.00	0.00	0.00
Apis mellifera	FJ	0.00	3.13	0.00	0.00	2.97	4.40	2.73	0.00	0.00	0.00	0.00	0.00	0.00	0.00	0.00	0.00	0.00
Nardostachys chinensis	GS	0.00	2.14	0.00	0.00	0.00	5.56	0.00	0.00	0.00	0.00	0.00	0.00	0.00	0.00	3.85	0.00	0.00
Pogostemon cablin	GHX	0.00	1.57	0.00	0.00	0.00	0.00	0.67	0.00	0.00	0.00	0.00	0.00	0.00	0.00	0.00	0.00	0.00
Magnolia officinalis	HP	0.00	2.71	0.00	0.00	0.00	3.54	0.00	0.00	0.00	0.00	0.00	2.97	0.00	0.00	0.00	0.00	0.00
Scutellaria baicalensis	HQN	0.00	0.00	0.00	0.00	1.64	0.00	0.84	0.00	0.00	0.00	0.00	0.00	0.00	0.00	0.00	0.00	0.00
Tribulus terrestris	JL	3.15	0.00	0.00	0.00	0.00	0.00	0.00	0.00	0.00	0.00	0.00	0.00	0.00	0.00	0.00	0.00	0.00
Lysimachia christinae	JQC	0.00	0.00	0.00	0.00	0.00	0.00	0.35	0.00	0.00	0.00	0.00	0.00	0.00	0.00	0.00	0.00	0.00
Cassia tora	JMZ	0.00	0.00	0.00	0.00	0.00	0.00	0.00	0.00	0.00	0.00	1.33	0.00	0.00	0.00	0.00	0.00	0.00
Sophora flavescens	KS	0.00	13.50	0.00	0.00	10.66	0.00	0.00	0.00	0.00	23.24	0.00	11.51	0.00	13.25	0.00	14.21	20.59
Ephedra sinica	MH	0.00	0.00	0.00	0.00	0.00	0.00	0.35	0.00	0.00	0.00	0.00	0.00	0.00	0.00	0.00	0.00	0.00
Ophiopogon japonicus	MD	0.00	0.00	0.00	2.88	0.00	0.00	0.00	0.00	11.40	0.00	0.00	4.10	0.00	0.00	0.00	0.00	0.00
Aucklandia lappa	MX	0.00	0.00	0.00	2.45	0.00	3.17	0.00	0.00	0.00	0.00	0.00	0.00	0.00	0.00	3.82	0.00	0.00
Equisetum hiemale	MZ	0.00	0.00	0.00	0.00	0.00	0.00	0.35	0.00	0.00	0.00	0.00	0.00	0.00	0.00	0.00	0.00	0.00
Artemisia annua	QH	0.00	4.92	0.00	0.00	0.00	0.00	2.05	0.00	0.00	0.00	0.00	0.00	0.00	0.00	0.00	0.00	3.25
Panax ginseng	RS	0.00	0.00	0.00	0.00	0.00	0.00	0.35	0.00	0.00	0.00	0.00	0.00	0.00	0.00	0.00	0.00	0.00
Morus alba	SBP	0.00	0.00	0.00	3.02	0.00	0.00	0.00	0.00	0.00	0.00	0.00	9.63	0.00	7.80	0.00	0.00	16.06
Morus alba	SY	0.00	0.00	0.00	2.79	0.00	0.00	0.00	0.00	0.00	0.00	0.00	3.72	0.00	0.00	0.00	5.85	0.00
Hippophae rhamnoides	SJ	0.00	0.00	0.00	0.00	0.00	0.00	0.35	0.00	0.00	0.00	0.00	0.00	0.00	0.00	0.00	0.00	0.00

Sophora tonkinensis	SDG	2.20	0.00	0.00	0.00	0.00	0.00	0.00	0.00	0.00	0.00	0.00	0.00	0.00	0.00	0.00	0.00	0.00
Kaempferia galanga	SN	0.00	0.00	0.00	0.00	0.00	0.00	0.35	0.00	0.00	0.00	0.00	0.00	0.00	0.00	0.00	0.00	0.00
Caesalpinia sappan	SM	0.00	0.00	0.00	0.00	0.00	0.00	0.69	0.00	0.00	0.00	0.00	0.00	0.00	0.00	0.00	0.00	2.12
Asparagus cochinchinensis	TD	0.00	0.92	0.00	0.00	0.00	0.00	0.00	0.00	0.00	0.00	0.00	0.00	0.00	0.00	0.00	0.00	0.00
Gastrodia elata	TM	0.00	0.00	0.00	0.00	0.00	1.15	0.00	0.00	0.00	0.00	0.00	0.00	0.00	0.00	0.00	0.00	0.00
Cuscuta australis	TSZ	0.00	0.00	0.00	0.00	0.00	0.00	0.35	0.00	0.00	0.00	0.00	0.00	0.00	0.00	0.00	0.00	0.00
Schisandra chinensis	WWZ	0.00	1.07	0.00	0.00	0.00	0.00	0.00	0.00	0.00	0.00	0.00	0.00	0.00	0.00	0.00	0.00	0.00
Evodia rutaecarpa	WZY	0.00	0.00	0.00	0.00	1.11	0.00	0.00	0.00	0.00	0.00	0.00	0.00	5.32	0.00	0.00	0.00	0.00
Cyperus rotundus	XF	0.00	0.00	0.00	0.00	0.00	1.65	0.00	0.00	0.00	0.00	2.26	0.00	0.00	0.00	0.00	0.00	0.00
Crocus sativus	XHH	0.00	0.00	0.00	0.00	0.00	0.00	0.35	0.00	0.00	0.00	0.00	0.00	0.00	0.00	0.00	0.00	0.00
Siegesbeckia orientalis	XXC	0.00	0.00	0.00	1.43	0.00	0.00	0.00	0.00	0.00	0.00	0.00	0.00	0.00	0.00	2.81	0.00	0.00
Inula britannica	XFH	0.00	0.00	0.00	0.00	0.00	0.00	0.35	0.00	0.00	0.00	0.00	0.00	0.00	0.00	0.00	0.00	0.00
Epimedium brevicornum	YYH	0.00	0.00	0.00	0.00	0.00	0.00	0.00	2.69	0.00	0.00	0.00	0.00	0.00	0.00	0.00	0.00	0.00
Alpinia oxyphylla	YZX	0.00	0.00	0.00	0.00	1.49	2.20	0.00	0.00	0.00	0.00	0.00	0.00	0.00	0.00	0.00	0.00	0.00
Curcuma longa	YJ	0.00	0.98	0.00	0.00	0.00	0.00	0.00	0.00	0.00	0.00	0.00	0.00	0.00	0.00	0.00	0.00	0.00
Arnebia euchroma	ZC	0.00	2.21	0.00	0.00	2.61	0.00	0.00	8.19	0.00	0.00	0.00	0.00	0.00	0.00	0.00	0.00	0.00

Supplementary Table S17. HTF of active herbs in XFZY group against 17 viral proteins (top 50% cutoff).

Herb	PR	RT1	RT2	IN	NC	CA1	CA2	MA	Nef1	Nef2	Vpr	gp120	gp41	CCR5	CXCR4	CycT1	ELOC
Bupleurum chinense	0.00	0.00	0.00	0.00	0.00	2.95	0.00	0.00	0.00	4.55	0.00	0.00	0.00	0.00	0.00	0.00	0.00
Ligusticum wallichii	0.00	13.11	0.00	0.00	60.06	25.97	0.00	0.00	0.00	0.00	20.72	0.00	0.00	0.00	0.00	0.00	0.00
Paeonia rubra	22.38	10.53	0.00	0.00	0.00	5.55	0.00	0.00	0.00	0.00	0.00	0.00	0.00	0.00	0.00	0.00	0.00
Angelica sinensis	0.00	0.00	0.00	25.05	0.00	7.39	0.00	0.00	0.00	0.00	5.83	25.20	0.00	0.00	0.00	0.00	25.10
Rehmannia glutinosa	0.00	0.00	0.00	0.00	4.15	10.92	0.00	0.00	0.00	0.00	0.00	0.00	0.00	0.00	0.00	0.00	0.00
Achyranthes bidentate	0.00	6.55	0.00	0.00	0.00	0.00	0.00	0.00	0.00	4.55	0.00	0.00	0.00	0.00	0.00	0.00	0.00

Supplementary Table S18. The Latin name, Chinese name and compound number of TCMHD herbs.

Latin name	Chinese name	Compound number	Latin Name	Chinese Name	Compound number
Artemisia argyi	aiye	34	***Gentiana scabra***	longdan	8
Ferula assafoetida	awei	17	***Aloe barbadensis***	luhui	31
Croton tiglium	badou	33	***Apocynum venetum***	luobumaye	9
Stemona tuberosa	baibu	23	***Trachelospermum jasminoides***	luoshiteng	10
Ginkgo biloba	baiguo	35	***Portulaca oleracea***	machixian	7
Bletilla striata	baiji	25	***Ephedra sinica***	mahuang	21
Chelidonium majus	baiqucai	28	***Ophiopogon japonicus***	maidong	32
Paeonia lactiflora	baishao	33	***Hordeum vulgare***	maiya	11
Pulsatilla chinensis	baitouweng	14	***Vitex trifolia***	manjingzi	29
Cynanchum atratum	baiwei	10	***Rhododendron dauricum***	manshanhong	19
Dictamnus dasycarpus	baixianpi	22	***Strychnos nuxvomica***	maqianzi	28
Angelica dahurica	baizhi	36	***Rosa rugosa***	meiguihua	47
Atractylodes macrocephala	baizhu	10	***Prunus mume***	meihua	3
Illicium verum	bajiaohuixiang	17	***Dryopteris crassirhizoma***	mianmaguanzhong	12
Morinda officinalis	bajitian	10	***Commiphora myrrha***	moyao	11
Isatis indigotica	banlangen	21	***Paeonia suffruticosa***	mudanpi	7
Pinellia ternata	banxia	14	***Oroxylum indicum***	muhudie	4
Syringa reticulata	baomazipi	8	***Vitex negundo***	mujingye	12
Menispermum dauricum	beidougen	35	***Akebia quinata***	mutong	10
Glehnia littoralis	beishashen	41	***Aucklandia lappa***	muxiang	53
Piper longum	biba	12	***Equisetum hiemale***	muzei	15
Areca catechu	binlang	23	***Daucus carota***	nanheshi	15
Mentha haplocalyx	bohe	11	***Schisandra sphenanthera***	nanwuweizi	12
Psoralea corylifolia	buguzhi	29	***Rhododendron molle***	naoyanghua	16
Atractylodes lancea	cangzhu	29	***Arctium lappa***	niubangzi	10
Platycladus orientalis	cebaiye	19	***Achyranthes bidentata***	niuxi	12

Bupleurum chinense	chaihu	49	***Ligustrum lucidum***	nvzhenzi	20
Citrus reticulata	chenpi	41	***Eupatorium fortunei***	peilan	27
Aquilaria sinensis	chenxiang	28	***Fritillaria ussuriensis***	pingbeimu	10
Plantago asiatica	cheqiancao	12	***Eriobotrya japonica***	pipaye	19
Paeonia lactiflora	chishao	9	***Taraxacum officinale***	pugongying	26
Vigna angularis	chixiaodou	5	***Typha angustata***	puhuang	17
Dioscorea nipponica	chuanshanlong	7	***Notopterygium incisum***	qianghuo	44
Aconitum carmichaeli	chuanwu	25	***Peucedanum praeruptorum***	qianhu	25
Andrographis paniculata	chuanxinlian	24	***Euphorbia lathyris***	qianjinzi	10
Ligusticum chuanxiong	chuanxiong	58	***Pharbitis nil***	qianniuzi	12
Ailanthus altissima	chunpi	15	***Sinomenium acutum***	qingfengteng	13
Acanthopanax senticosus	ciwujia	17	***Artemisia annua***	qinghao	104
Rheum officinale	dahuang	31	***Gentiana macrophylla***	qinjiao	13
Angelica sinensis	danggui	51	***Dianthus superbus***	qumai	8
Codonopsis pilosula	dangshen	57	***Lonicera japonica***	rendongteng	3
Salvia miltiorrhiza	danshen	65	***Panax ginseng***	renshen	78
Oryza sativa	daoya	20	***Cistanche deserticola***	roucongrong	27
Isatis indigotica	daqingye	15	***Myristica fragrans***	roudoukou	11
Allium sativum	dasuan	30	***Cinnamomum cassia***	rougui	11
Sargentodoxa cuneata	daxueteng	6	***Boswellia carterii***	ruxiang	14
Gleditsia sinensis	dazaojiao	4	***Saururus chinensis***	sanbaicao	19
Ziziphus jujuba	dazao	34	***Morus alba (cortex)***	sangbaipi	47
Juncus effuses	dengxincao	13	***Morus alba (mulberry)***	sangshen	10
Kadsura interior	dianjixueteng	17	***Morus alba (folium)***	sangye	42
Rehmannia glutinosa	dihuang	67	***Morus alba (ramulus)***	sangzhi	16
Eugenia caryophyllata	dingxiang	19	***Panax notoginseng***	sanqi	51
Sanguisorba officinalis	diyu	31	***Hippophae rhamnoides***	shaji	48
Cordyceps sinensis	dongchongxiacao	19	***Sophora tonkinensis***	shandougen	20

Rabdosia rubescens	donglingcao	31	***Phytolacca acinosa***	shanglu	11
Amomum kravanh	doukou	7	***Kaempferia galanga***	shannai	9
Angelica pubescens	duhuo	41	***Dioscorea opposita***	shanyao	21
Eucommia ulmoides	duzhong	34	***Crataegus pinnatifida***	shanzha	18
Saposhnikovia divaricata	fangfeng	24	***Cornus officinalis***	shanzhuyu	30
Stephania tetrandra	fangji	17	***Astragalus complanatus***	shayuanzi	5
Cassia acutifolia	fanxieye	8	***Cnidium monnieri***	shechuangzi	20
Dioscorea hypoglauca	fenbixie	10	***Belamcanda chinensis***	shegan	16
Apis mellifera	fengjiao	10	***Zingiber officinale***	shengjiang	77
Poria cocos	fuling	33	***Cimicifuga foetida***	shengma	10
Aconitum carmichaeli	fuzi	39	***Lycopodium japonicum***	shenjincao	17
Glycyrrhiza uralensis	gancao	84	***Dendrobium nobile***	shihu	20
Zingiber officinale	ganjiang	29	***Punica granatum***	shiliupi	11
Nardostachys chinensis	gansong	29	***Silybum marianum***	shuifeiji	13
Euphorbia kansui	gansui	42	***Luffa cylindrica***	sigualuo	9
Ligusticum sinense	gaoben	11	***Ziziphus jujuba***	suanzaoren	21
Alpinia officinarum	gaoliangjiang	22	***Caesalpinia sappan***	sumu	28
Pueraria lobata	gegen	20	***Aesculus chinensis***	suoluozi	10
Lycium chinense	gouqizi	41	***Santalum album***	tanxiang	24
Uncaria rhynchophylla	gouteng	31	***Asparagus cochinchinensis***	tiandong	14
Trichosanthes kirilowii	gualou	30	***Trichosanthes kirilowii***	tianhuafen	9
Pogostemon cablin	guanghuoxiang	23	***Gastrodia elata***	tianma	18
Phellodendron amurense	guanhuangbai	22	***Cinnamomum camphora***	tianranbingpian	12
Hypericum perforatum	guanyejinsitao	10	***Hyoscyamus niger***	tianxianzi	24
Cinnamomum cassia	guizhi	10	***Tetrapanax papyriferus***	tongcao	14
Piper kadsura	haifengteng	30	***Bolbostemma paniculatum***	tubeimu	12
Eclipta prostrata	hanmolian	11	***Smilax glabra***	tufuling	9
Albizzia julibrissin	hehuanpi	22	***Pseudolarix amabilis***	tujingpi	24

Glycine max	heidou	21	***Cuscuta australis***	tusizi	8
Carpesium abrotanoides	heshi	7	***Vaccaria segetalis***	wangbuliuxing	11
Polygonum multiflorum	heshouwu	13	***Claviceps purpurea***	weilingxian	8
Juglans regia	hetaoren	26	***Schisandra chinensis***	wuweizi	68
Nelumbo nucifera	heye	13	***Lindera aggregata***	wuyao	17
Terminalia chebula	hezi	13	***Evodia rutaecarpa***	wuzhuyu	47
Alpinia galanga	hongdoukou	13	***Prunella vulgaris***	xiakucao	32
Carthamus tinctorius	honghua	32	***Cyperus rotundus***	xiangfu	18
Magnolia officinalis	houpu	44	***Periploca sepium***	xiangjiapi	36
Sophora japonica	huaihua	7	***Citrus medica***	xiangyuan	8
Zanthoxylum bungeanum	huajiao	11	***Curculigo orchioides***	xianmao	17
Coptis chinensis	huanglian	12	***Foeniculum vulgare***	xiaohuixiang	11
Scutellaria baicalensis	huangqin	44	***Podophyllum emodii***	xiaoyelian	15
Astragalus membranaceus	huangqi	16	***Rubia cordifolia***	xicao	42
Fritillaria hupehensis	hubeibeimu	10	***Allium macrostemon***	xiebai	19
Picrorhiza scrophulariaeflora	huhuanglian	12	***Crocus sativus***	xihonghua	42
Piper nigrum	hujiao	55	***Magnolia denudata***	xinyi	13
Viscum coloratum	hujisheng	5	***Siegesbeckia orientalis***	xixiancao	27
Trigonella foenumgraecum	huluba	40	***Asarum sieboldii***	xixin	22
Cannabis sativa	huomaren	9	***Panax quinquefolium***	xiyangshen	25
Polygonum cuspidatum	huzhang	25	***Inula britannica***	xuanfuhua	15
Dalbergia odorifera	jiangxiang	34	***Dipsacus asperoides***	xuduan	12
Platycodon grandiflorum	jiegeng	12	***Brucea javanica***	yadanzi	58
Brassica juncea	jiezi	16	***Cissampelos pareira***	yahunu	16
Celosia cristata	jiguanhua	10	***Corydalis yanhusuo***	yanhusuo	31
Abrus cantoniensis	jigucao	11	***Chrysanthemum indicum***	yejuhua	9
Tribulus terrestris	jili	15	***Eupatorium lindleyanum***	yemazhui	20
Inula japonica	jinfeicao	23	***Stauntonia chinensis***	yemugua	6

Schizonepeta tenuifolia	jingjie	22	***Leonurus artemisia***	yimucao	30
Ajuga decumbens	jingucao	21	***Stellaria dichotoma***	yinchaihu	14
Conyza blinii	jinlongdancao	8	***Artemisia capillaries***	yinchen	54
Lysimachia christinae	jinqiancao	3	***Papaver somniferum***	yingsuke	13
Psammosilene tunicoides	jintiesuo	8	***Ginkgo biloba***	yinxingye	20
Rosa laevigata	jinyingzi	12	***Epimedium brevicornum***	yinyanghuo	14
Lonicera japonica	jinyinhua	32	***Solidago decurrens***	yizhihuanghua	8
Murraya paniculata	jiulixiang	60	***Alpinia oxyphylla***	yizhi	26
Centella asiatica	jixuecao	7	***Daphne genkwa***	yuanhua	11
Selaginella tamariscina	juanbai	12	***Polygala tenuifolia***	yuanzhi	29
Cassia tora	juemingzi	15	***Phyllanthus emblica***	yuganzi	44
Chrysanthemum morifolium	juhua	10	***Curcuma longa***	yujin	22
Cichorium intybus	juju	11	***Houttuynia cordata***	yuxingcao	23
Tussilago farfara	kuandonghua	18	***Echinops grijsii***	yuzhouloulu	9
Corydalis bungeana	kudiding	11	***Acorus calamus***	zangcangpu	28
Melia azedarach	kulianpi	31	***Alisma orientale***	zexie	29
Picrasma quassioides	kumu	76	***Fritillaria thunbergii***	zhebeimu	31
Sophora flavescens	kushen	74	***Anemarrhena asphodeloides***	zhimu	23
Prunus armeniaca	kuxingren	22	***Citrus aurantium***	zhishi	12
Picria felterrae	kuxuanshen	9	***Valeriana jatamansii***	zhizhuxiang	17
Capsicum annuum	lajiao	33	***Gardenia jasminoides***	zhizi	27
Zanthoxylum nitidum	liangmianzhen	12	***Panax pseudoginseng***	zhujieshen	6
Forsythia suspensa	lianqiao	27	***Arnebia euchroma***	zicao	28
Nelumbo nucifera (plumule)	lianzixin	9	***Peucedanum decursivum***	zihuaqianhu	17
Nelumbo nucifera (seed)	lianzi	10	***Perilla frutescens***	zisuye	10
Ganoderma lucidum	lingzhi	102	***Aster tataricus***	ziwan	17
